# Glutathione Peroxidase 3 induced mitochondria-mediated apoptosis via AMPK /ERK1/2 pathway and resisted autophagy-related ferroptosis via AMPK/mTOR pathway in hyperplastic prostate

**DOI:** 10.1186/s12967-023-04432-9

**Published:** 2023-08-26

**Authors:** Yan Li, Yongying Zhou, Daoquan Liu, Zhen Wang, Jizhang Qiu, Junchao Zhang, Ping Chen, Guang Zeng, Yuming Guo, Xinghuan Wang, Michael E. DiSanto, Xinhua Zhang

**Affiliations:** 1 Department of Urology, Zhongnan Hospital of Wuhan University, 169 Donghu Road, Wuhan, 430071 People’s Republic of China; 2 Department of Surgery and Biomedical Sciences, Cooper Medical School of Rowan University, Camden, NJ USA

**Keywords:** Glutathione peroxidase 3, Benign prostatic hyperplasia, Mitochondria-mediated apoptosis, Autophagy-related ferroptosis, AMPK/ERK1/2 pathway, AMPK/mTOR pathway

## Abstract

**Background:**

Benign prostatic hyperplasia (BPH) is a common disease in elderly men, mainly resulted from an imbalance between cell proliferation and death. Glutathione peroxidase 3 (GPX3) was one of the differentially expressed genes in BPH identified by transcriptome sequencing of 5 hyperplastic and 3 normal prostate specimens, which had not been elucidated in the prostate. This study aimed to ascertain the mechanism of GPX3 involved in cell proliferation, apoptosis, autophagy and ferroptosis in BPH.

**Methods:**

Human prostate tissues, GPX3 silencing and overexpression prostate cell (BPH-1 and WPMY-1) models and testosterone-induced rat BPH (T-BPH) model were utilized. The qRT-PCR, CCK8 assay, flow cytometry, Western blotting, immunofluorescence, hematoxylin and eosin, masson’s trichrome, immunohistochemical staining and transmission electron microscopy analysis were performed during in vivo and in vitro experiments.

**Results:**

Our study indicated that GPX3 was localized both in the stroma and epithelium of prostate, and down-regulated in BPH samples. Overexpression of GPX3 inhibited AMPK and activated ERK1/2 pathway, thereby inducing mitochondria-dependent apoptosis and G0/G1 phase arrest, which could be significantly reversed by MEK1/2 inhibitor U0126 preconditioning. Moreover, overexpression of GPX3 further exerted anti-autophagy by inhibiting AMPK/m-TOR and up-regulated nuclear factor erythroid 2-related factor 2 (Nrf2)/glutathione peroxidase 4 (GPX4, mitochondrial GPX4 and cytoplasmic GPX4) to antagonize autophagy-related ferroptosis. Consistently, GPX3 deficiency generated opposite changes in both cell lines. Finally, T-BPH rat model was treated with GPX3 indirect agonist troglitazone (TRO) or GPX4 inhibitor RAS-selective lethal 3 (RSL3) or TRO plus RSL3. These treatments produced significant atrophy of the prostate and related molecular changes were similar to our in vitro observations.

**Conclusions:**

Our novel data manifested that GPX3, which was capable of inducing apoptosis via AMPK/ERK1/2 pathway and antagonizing autophagy-related ferroptosis through AMPK/m-TOR signalling, was a promising therapeutic target for BPH in the future.

**Supplementary Information:**

The online version contains supplementary material available at 10.1186/s12967-023-04432-9.

## Background

Benign prostatic hyperplasia (BPH) is the general non-malignant disease in elderly men and its prevalence rises with age, up to 50% in men over 50 years old [1]. Lower urinary tract symptoms (LUTS), often secondary to BPH, are worrisome complication that place significant encumbrance on patients’ quality of life [2]. The increase in the number of periurethral epithelial and stromal cells characterizes BPH histopathologically due to the maladjustment of cell proliferation and death [3]. Although the molecular mechanism of BPH have been extensively studied, including a disequilibrium of sex hormone [4], stromal-epithelial interactions [5], cytokins [6], autoimmune [7] and oxidative stress (OS) [8], the exact pathogenesis remains unclear. In recent years, with the continuous progress of experimental technology, from PCR to a new generation of high-throughput sequencing to multi-omics, more and more molecular mechanisms related to BPH will be elucidated. Among them, the identification of differentially expressed genes (DEGs) through transcriptome sequencing (RNA-seq) to explore the molecular mechanism, the diagnostic markers and therapeutic targets have become the research directions of many diseases. So did with BPH. Indeed, we performed RNA-seq of 5 hyperplastic and 3 normal prostate specimens and found that there was a number of DEGs between hyperplastic prostate and normal prostate, of which the up-expressed matrix-remodeling associated 5 (MXRA5) [9], C-X-C motif chemokine ligand 13 (CXCL13) [10], bone morphogenetic protein 5 (BMP5) [11] and neural epidermal growth factor-like like 2 (NELL2) [12], and the down-expressed glutathione peroxidase 3 (GPX3) ranked at the top and most of them have been reported. However, the role and function of GPX3 gene in the development of BPH and its potential molecular mechanisms have not been elucidated.

The glutathione peroxidase family is composed of eight isozymes, and selenocysteine is present in five of them (GPX1–4, GPX6) [13]. GPX3 encodes a 24 kDa 1homotetramer protein spanning five exons on chromosome 5q32 [14, 15]. In addition, plasma GPX3 is the dominant extracellular GPX isoform, and the kidneys are the major tissues that express it [16]. Functionally, GPX3 exerts its antioxidant effect through the reduction of H_2_O_2_ and hydroperoxides by consuming glutathione (GSH) [17]. And it has been observed involved a number of diseases, including cancers of the gastric [18], ovarian [19] and prostate [20], where it showed suppressive effect.

Mitochondria are not only the energy metabolism center of eukaryotic cells, but also the main source of intracellular reactive oxygen species (ROS) [21]. Also, mitochondria regulates the type of cell death (e.g. apoptosis, autophagy and ferroptosis) through a variety of mechanisms [22, 23]. For example, the release of mitochondrial cytochrome c (Cyto-C) could initiate endogenous apoptosis with concomitant depolarization of the mitochondrial membrane potential (MMP) [24]. However, the role of GPX3 related redox metabolism, especially its association with mitochondria on cell proliferation and cell death in BPH has not been well illustrated.

Ferroptosis was first reported in 2012 [25]. And it’s a form of programmed cell death (PCD) that occurs as a result of iron-dependent oxidative reactions different in both biochemistry and morphology from others (apoptosis, necrosis and autophagy) [26]. During ferroptosis, the lipids in the cyto-membranes undergo superfluous oxidative damage [27]. This process is often accompanied by up-regulation of intracellular labile iron, transferrin and malondialdehyde (MDA), and down-regulation of GSH and anti-ferroptosis systems [28]. It has been shown that the development of BPH is accompanied by abnormally elevated OS and ROS surplus [8], which can cause cellular dysfunction and tissue damage [29]. There have been many studies on ferroptosis in several of tumors including prostate cancer [30]. But whether and how ferroptosis affects BPH has not been reported.

Autophagy is a natural, regulated PCD behavior. The mechanism is through a variety of autophagy-related proteins (including Beclin1 and LC3B, which are the initial protein and structural components of autophagosomes, respectively) to form double membrane vesicles to engulf damaged proteins or organelles and bind to lysosomes [31]. Functionally, autophagy exists in the life process as a survival mechanism of stress response [31]. Although autophagy and ferroptosis are mechanistically and morphologically distinct cell death pathways, a growing number of recent studies have reported significant interactions between them [32–34]. In particular, elucidating the relationship between autophagy and ferroptosis in BPH, which has traditionally used apoptosis as the main PCD mode, will provide a deeper understanding of the regulation of cell death in BPH.

In our current study, we analysed the expression of GPX3 and PCD patterns (apoptosis, autophagy and ferroptosis) in human normal and hyperplastic prostates. Furthermore, we established GPX3 silencing and up-regulation prostate cell models to investigate the effects and mechanisms of GPX3 on cell proliferation, apoptosis, autophagy and ferroptosis level of prostate cells in vitro. Finally, we validated the role of GPX3 in vivo via testosterone-BPH (T-BPH) rat model.

## Methods

Cell lines obtained and cultured, CCK-8 assay, total RNA extraction, RNA reverse transcription, qRT-PCR analysis, Western blot analysis, hematoxylin and eosin (H&E) staining and masson’s trichrome staining analysis were performed as we previously described [35, 36]. Primer sequences were summarized in Additional file 1: Table S1. The primary and secondary antibodies were summarized in Additional file 2: Table S2 and Additional file 3: Table S3. All experiments were repeated at least 3 times.

### Cell immunofluorescence staining

For cell immunofluorescence microscopy, cells were seeded on 12 mm coverslips and washing by ice-cold (PBS, pH = 7.4). The coverslips were then fixed with 4% paraformaldehyde (PFA) for 30 min, followed by 0.1% Triton X-100 incubation, and then blocked in goat serum for 30 min at room temperature. Afterward, they were incubated with primary antibody (listed in Additional file 2: Table S2) at room temperature for 2 h, washed with PBS, and incubated with Cy3- or FITC-labeled secondary antibody (listed in Additional file 3: Table S3) for 1 h. Nuclei were labeled with DAPI (4ʹ,6-diamidino-2-phenylindole) (2 μg/ml). Visualization was done with a laser scanning confocal microscope (Olympus, Tokyo, Japan).

### Tissue immunofluorescence staining

Human prostate tissues were sectioned in 10 μm-thick slices and thawed, mounted onto glass slides using a cryostat (Leica CM 1850, Wetzlar, Germany), air-dried, and fixed for 10 min in ice-cold acetone. Slides were washed in PBS and incubated for 2 h in a mixture of PBS supplemented with 0.2% Triton X-100 and 0.1% bovine serum albumin, followed by incubation overnight with the primary antibodies (listed in Additional file 2: Table S2). The secondary antibodies (listed in Additional file 3: Table S3) employed to visualize the localization of primary antibodies were Cy3-conjugated goat anti-rabbit IgG (1:1000). DAPI was used for staining the nucleus. Visualization was done with a Laser Scanning Confocal Microscope (Olympus, Tokyo, Japan).

### Immunohistochemical(IHC) staining

Human and rat prostate tissues fixed in 10% neutral buffered formalin for 48 h were routinely processed for paraffin embedding. Samples were sectioned at 5 μm and deparaffinized in xylene followed by descending grades of ethanol (100, 95, 70, 30%). Antigen retrieval was performed in 10 mM sodium citrate buffer at pH 6.0, heated to 96 °C, for 30 min, followed by proteinase K treatment for 10 min. Endogenous peroxidase activity was quenched using 3% hydrogen peroxide in PBS for 15 min. Blocking was performed by incubating sections in 5% normal donkey serum with 2% BSA for 1 h. The sections were stained by routine immunohistochemical (IHC) methods, using horse radish peroxidase polymer conjugate (Invitrogen), to localize the antibody bound to antigen, with diaminobenzidine as the final chromogen. All immunostained sections were lightly counterstained with Hematoxylin. The primary antibodies (information listed in Additional file 2: Table S2) to target proteins were incubated for 1 h at room temperature. Slides were evaluated for immunostaining by light microscopy. All specimens were stained with IHC. For each field, integrated optical density (IOD) was calculated using Image-J software. The mean density was calculated by IOD/area and the average values were used for target protein expression quantitative analyses.

### Public data acquiring

The gene expression profiling dataset GSE119195 was obtained from GEO (https://www.ncbi.nlm.nih.gov/geo/) of the NCBI and the mRNA expression profiles of GPX3 between human hyperplastic prostate and normal prostate specimens were analyzed.

### Animals and tissues

In total, 40 male Sprague–Dawley rats (6 weeks old) were randomly allocated into five groups (n = 8 per group): control group, corn oil (MedChemExpress, China) injection [subcutaneously (s.c.)] + dimethyl sulfoxide (DMSO) (MedChemExpress, China) [intraperitoneally (i.p.)] + DMSO [intragastrically (i.g.)]; T group, T (testosterone propionate, Sigma-Aldrich, St. Louis, MO) (2 mg/day)/corn oil injection (s.c.) + DMSO (i.p.) + DMSO (i.g.); T + RSL3 group, T injection (s.c.) + RSL3 (RAS-selective lethal 3, induces ferroptosis by inhibiting GPX4, MedChemExpress, China) (10 mg/kg/day, i.p.) + DMSO (i.g.); T + TRO group, T injection (s.c.) + DMSO (i.p.) + TRO [troglitazone is a ligand of peroxisome proliferator-activated receptor γ (PPARγ) as the GPX3 agonist, MedChemExpress, China] (15 mg/kg/day, i.g.); T + RSL3 + TRO group, T (s.c.) + RSL3 (i.p.) + TRO (i.g.). On day 28, ventral prostates and seminal vesicles were harvested and weighed. All surgical procedures were performed under anesthesia by i.p. injection of pentobarbital sodium (35 mg/kg; Abbott Laboratory, Chicago, IL). All animal protocols were approved by the Medical Ethics Committee for Experiments at Zhongnan Hospital of Wuhan University before the experiments were conducted.

Four prostate samples from young brain-dead men (mean age 25.2 ± 4.4 years old) undergoing organ donation were obtained as normal ones. Eight specimens of hyperplastic prostate (mean age 70.0 ± 7.5 years old) were came from the patients who underwent transurethral resection prostate. Postoperative prostate pathology examination revealed BPH. Human samples were collected after approval of Hospital Committee for Investigation in Humans and after receiving written informed consent from each patient or relative. All human studies were conducted in accordance with the principles of the Declaration of *Helsinki*.

### Knockdown and overexpression of GPX3 in human prostate cells

GPX3-target specific small interfering RNA (siRNA) was synthesized by Genepharma Ltd. in Suzhou, China. When cells were 30–50% confluent in 6-well culture plates, the medium was replaced with fresh one 30 min before transfection. Following the instructions of Genepharma Ltd. in Suzhou, China, the transfection media were prepared and incubated for 10 min. Subsequently, we added 200 μL of lipofectamine (Genepharma Ltd. in Suzhou, China) complex solution to each well and then incubated for 6 h. After that the medium was replaced with fresh medium and incubated for 48–72 h. The sense sequences of si-GPX3 are listed in Additional file 4: Table S4.

GPX3 complementary DNA (cDNA) were PCR amplified from a cDNA library of human cell line and then a homologous recombination vector was constructed by cloning it into an empty FlagpcDNA3 vector. When cells were 40–60% confluent in 6-well culture plates, the medium was replaced with fresh one 30 min before transfection. Following the instructions of Genepharma Ltd. in Suzhou, China, the transfection media were prepared and incubated for 10 min. Subsequently, we added 200 μL of lipofectamine (Genepharma Ltd. in Suzhou, China) complex solution to each well and then incubated for 6 h. After that the medium was replaced with fresh medium and incubated for 48–72 h.

### Drug treatment

In order to deactivate ERK1/2, prostate cells were pretreated with 10 μM MEK1/2 inhibitor U0126 (MedChemExpress, China) for 24 h prior to plasmid transfection. And, prostate cells were pretreated with RSL3 (MedChemExpress, China) at 0.25 μM, 0.5 μM, 1 μM, 2 μM or 4 μM for 24 h to activate ferroptosis, isosilybin B (MedChemExpress, China) at 20 μM, 40 μM, 60 μM, 80 μM or 100 μM for 24 h to activate apoptosis, Rapamycin (Rapa, MedChemExpress, China) at 0.5 nM, 1 nM, 2 nM, 4 nM or 8 nM for 24 h to activate autophagy, Chloroquine (CQ, MedChemExpress, China) at 10 μM, 20 μM, 30 μM, 40 μM or 50 μM for 24 h to inhibit autophagy, and GSK621 (MedChemExpress, China) at 5 μM, 10 μM, 20 μM, 30 μM or 40 μM for 24 h to activate AMPK. Meanwhile, equivalent amount of DMSO without drugs were added to the cells served as a control.

### Flow cytometry analysis

1 × 10^6^ cells were collected for cell cycle analysis. After centrifugation, cells were resuspended in the dark in PBS containing 50 µg/mL propidium iodide (Sigma-Aldrich, USA) and 0.1 mg/mL RNaseA (20 µg/mL PBS). Detection after 30 min incubation at 37 °C. Cells were collected 48 h after transfection for analysis of apoptosis, stained with FITC Annexin V Apoptosis Detection Kit I ( BD biosciences, USA), and analyzed by flow cytometry. Intracellular ROS levels were assessed using a fluorescent probe 2ʹ, 7ʹ -dichlorofluorescein diacetate (DCFH-DA Sigma-Aldrich, USA). After transfection and growth for 48 h, 10 μM DCFH-DA was added into 1 mL medium and incubated at 37 °C for 30 min. To detect MMP, cells were collected and stained with JC-1 (10 μg/mL, Invitrogen, Rockford, IL, USA) at 37 °C for 10–20 min.

### Isolation of cytosolic and mitochondrial fractions

A total of 2 × 10^7^ cells were collected and then mitochondrial separation agent (Beyotime Biotechnology Co., Ltd. in Wuhan, China) was added. The cell suspension was centrifuged at 700 g for 10 min at 4 °C, then the supernatant was transferred to a new 2 mL tube and centrifuged at 12,000 g for 15 min at 4 °C. The supernatant (cytoplasmic portion) was transferred to a new tube, and the granules contained isolated mitochondria.

### Measurement of intracellular iron, GSH and MDA

For the determination of iron levels, the collected cells and rat prostate tissue (10 mg) were homogenized in pre-cooled iron analysis buffer (Beyotime Biotechnology Co., Ltd. in Wuhan, China), and the supernatant was collected after centrifugation at 4 °C, 16,000 g for 10 min. The sample was incubated with an equal amount of analytical buffer at room temperature for 30 min. Then, 100 μL of the iron probe (Beyotime Biotechnology Co., Ltd. in Wuhan, China) was incubated in the dark at 25 °C for 60 min, and the absorbance was determined to be 593 nm.

Relative GSH concentration in cell and rat prostate tissue lysates was assessed using a Total Glutathione Quantification Kit (cat #T419; Dojingdo Laboratories). Samples were lysed using 5% 5‐sulfosalicylic acid solution. After centrifugation at 8000 g for 10 min, supernatants were collected and incubated with the substrate 5, 5′‐dithiobis (2‐nitrobenzoic acid) at 37 °C for 10 min, which produced yellow 5‐thio‐2‐nitrobenzoic acid. Subsequently, 20 μL substrate working solution was added and incubated for 10 min at room temperature. Absorbance was measured with a microplate reader at 412 nm.

Relative MDA concentration in cell and rat prostate tissue lysates was assessed using a MDA Quantification Kit (Beyotime Biotechnology Co., Ltd. in Wuhan, China) and MDA content was expressed as the relative absorbance of the sample at 532 nm.

### Transmission electron microscopy analysis

Cells were fixed with 2% paraformaldehyde and 2% glutaraldehyde in 0.1 mol/L phosphate buffer (pH 7.4) for 1 h, followed by postfixation for 6 h in 1% OsO_4_. After dehydration with graded alcohol (50, 70, 80, 90, 100%) solutions for 15 min each time, each sample was embedded in epoxy resin (Sigma-Aldrich, 45,359). The cut-thin sample (70 nm) was mounted on a copper mesh (Sigma-Aldrich, TEM-74357) and post-stained with 2% uranyl acetate and 1% lead citrate, dried, and analyzed with a transmission electron microscope (JEOL).

### Statistical analysis

Statistical analyzes were performed using SPSS statistical software, version 21.0 (IBM, Chicago, IL). The data were expressed as mean ± standard deviations from at least three independent experiments. Student’s two-tailed t-test was used to compare the means of two-group samples, and one-way ANOVA was applied for comparison of multiples groups. *p*-value < 0.05 was considered statistically significant. We set a single blind for the statisticians of the data in this study.

## Results

### The expression and localization of GPX3 in human prostate tissues and cell lines

**Fig. 1:**
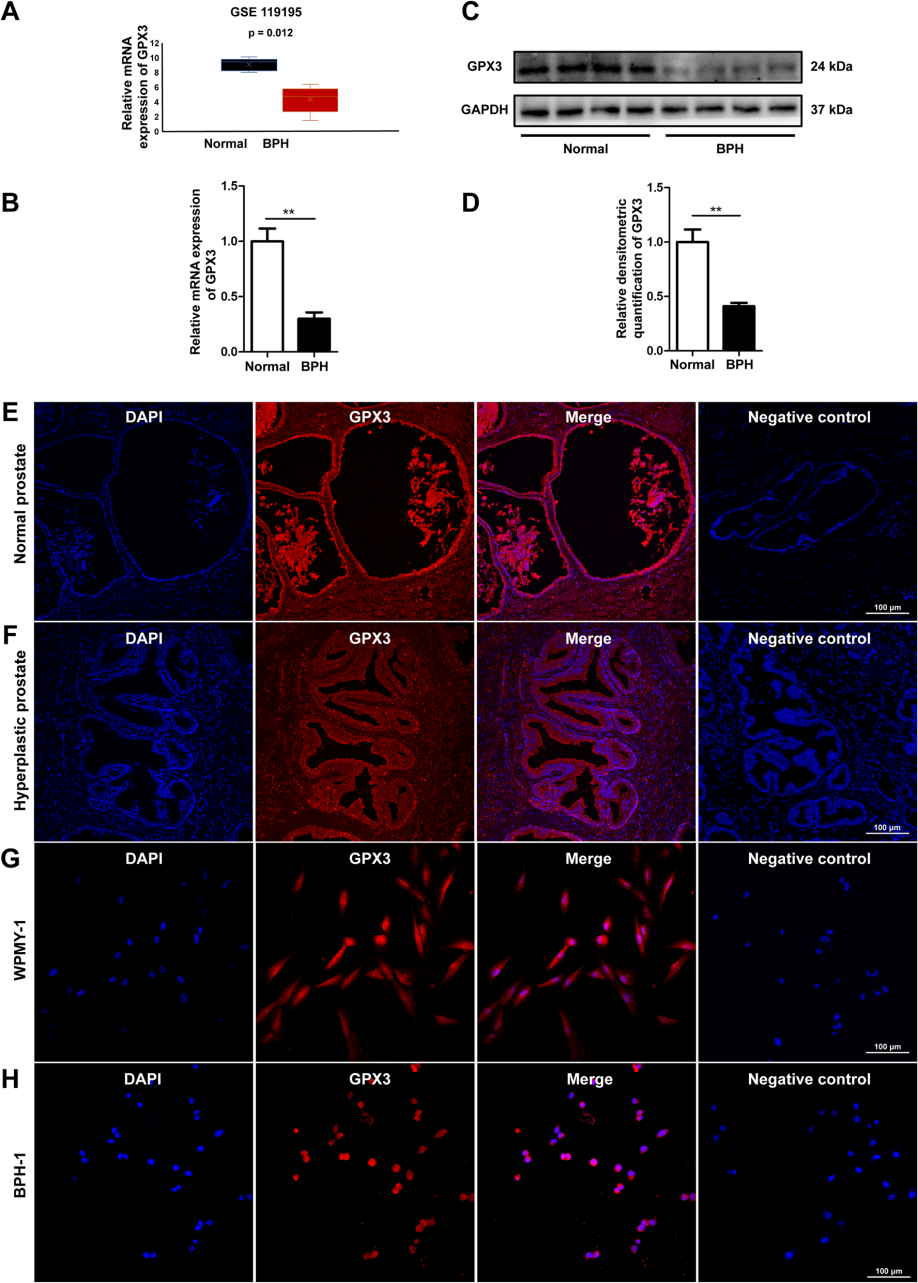
The localization and expression of GPX3 in human prostate tissues and cultured human prostate cells **A** The mRNA expression of GPX3 in BPH and normal prostate through GSE119195 dataset, which showed GPX3 expression was down-regulated at the transcriptional level in BPH. **B** qRT-PCR analysis demonstrating the expression level of GPX3 gene in BPH tissue (n = 8) and normal prostate tissue (n = 4), and the expression of GPX3 was decreased at mRNA level. **C** Immunoblot assay revealed the protein expression of GPX3 in normal prostate tissues (n = 4) and BPH tissues (n = 4), and the expression of GPX3 was decreased at protein level. **D** Relative densitometric quantification of GPX3 protein in BPH tissues versus normal ones. Immunofluorescence localization of GPX3 for normal prostate tissues (**E**) and BPH tissues (**F**), and GPX3 was localized both in epithelium and stroma. Immunofluorescence localization of GPX3 in WPMY-1 (**G**) and BPH-1 (**H**) cells. DAPI (blue) shows nuclear staining; Cy3-immunofluorescence (red) shows GPX3 protein. And negative control in each group omitting the primary antibody failed to stain. Sections of 5 hyperplastic and 3 normal human prostate samples were used for immunofluorescence experiments and cell immunofluorescence staining was repeated at least 3 times. And representative graphs were selected for figures. GAPDH is used as loading control. ^**^p < 0.01. The scale bars are 100 μm

Our microarray dataset (serial number: GSE119195) was used to analyze the differential expression of GPX3 transcriptome in 5 hyperplastic and 3 normal prostate specimens, which showed GPX3 expression was significantly down-regulated at the transcriptional level in hyperplastic prostates (Fig. 1A and Additional file 5: Fig S1). Similarly, tissues harvested from our institute were observed that the expression of GPX3 was decreased at both mRNA (n = 4 control/8 BPH samples) (Fig. 1B) and protein (n = 4 control/4 BPH samples) levels (Fig. 1C, D). Meanwhile, immunofluorescence staining of prostate tissue demonstrated that GPX3 was localized both in epithelium and stroma (Fig. 1E, F). Consistently, cell immunofluorescence staining demonstrated GPX3 was present both in cultured human stromal cell WPMY-1 (Fig. 1G) and epithelial cell BPH-1 (Fig. 1H).

### GPX3 silencing promoted the cell survival, shortened the cell G0/G1 phase, reduced mitochondria-mediated apoptosis and mitochondrial dysfunction of prostate cells via inhibiting ERK pathway activation

**Fig. 2:**
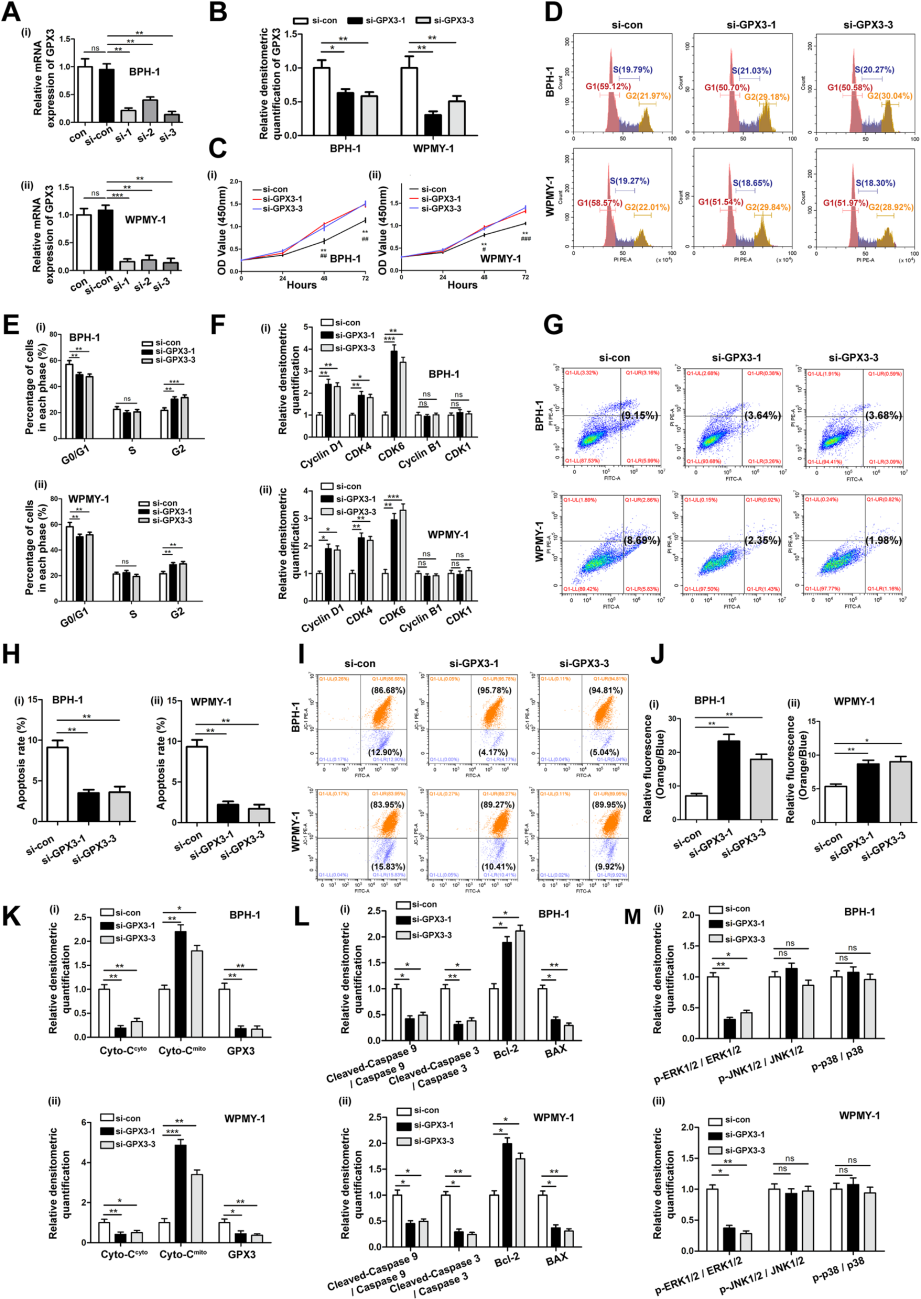
Effect of GPX3 knockdown on cell proliferation, cell cycle, cell apoptosis, mitochondrial membrane potential and MAPK signaling pathway in BPH-1 and WPMY-1 cells. Knockdown efficiency of GPX3 at the mRNA (**A**) and protein (**B**) levels with three different siRNA sequences in BPH-1 and WPMY -1 cells. **C** The cell viability of BPH-1 and WPMY -1 after knockdown of GPX3 at different time points by CCK-8 assay, and downregulation of GPX3 accelerated cell proliferation. ^*^: si-con vs. si-GPX3-1; ^#^: si-con vs. si-GPX3-3. **D** Flow cytometry analysis of cell cycle. **E** Histogram showing percentage of cell populations at different stages of the cell cycle (%), and G0/G1 phase was shortened in si-GPX3 transfected prostate cells with a corresponding increase of G2 phase but no significant change of S phase. **F** Relative densitometric quantification of Cyclin D1, CDK4, CDK6, Cyclin B1 and CDK1 protein in BPH-1 and WPMY-1 after knockdown of GPX3, and Cyclin D1, CDK4 and CDK6 underwent increase, while Cyclin B1 and CDK1 were no significant difference in prostate cells treated with si-GPX3. **G** Flow cytometry analysis of cell apoptosis. **H** Statistical analysis of apoptotic rate (%) showed GPX3 silencing inhibited the apoptosis of prostatic cells. **I** The mitochondrial membrane potential level of BPH-1 and WPMY-1 cells was examined by JC-1 staining. The scatter plot of the flow cytometry analysis shows the distribution of JC-1 aggregates (Orange) and JC-1 monomer (Blue) cell population. **J** Histogram calculated the relative ratio of Orange against Blue fluorescence, and silencing GPX3 contributed to more agminated MMP in prostate cells. **K** Relative densitometric quantification of Cyto-C protein in mitochondria or cytoplasm in BPH-1 and WPMY-1 after silence of GPX3, and GPX3 silencing led to a decrease in mitochondrial Cyto-C release. **L** Relative densitometric quantification of Cleaved-Caspase 9, Cleaved-Caspase 3, Bcl-2 and BAX protein in BPH-1 and WPMY-1 after knockdown of GPX3, and knockdown of GPX3 resulted in an increase in anti-apoptotic protein and a decrease in pro-apoptotic proteins. **M** Relative densitometric quantification of MAPK signaling pathway proteins in BPH-1 and WPMY-1 after knockdown of GPX3, and knockdown of GPX3 significantly inhibited the ERK1/2 pathway. ^*/#^*p* < 0.05, ^**/##^*p* < 0.01, ^***/###^*p* < 0.001 and ns means no significant difference

To investigate the functional activities of GPX3, the cell model of GPX3 silencing was constructed by three distinct si-GPX3s transfection. The mRNA level of GPX3 was more down-regulated by si-GPX3-1 and si-GPX3-3, which were selected for subsequent experiments (Fig. 2A). Meanwhile, GPX3 was silenced at the protein level after transfection of prostate cells with si-GPX3-1 and si-GPX3-3 (Fig. 2B and Additional file 6: Fig. S2A). Cell proliferation was detected with CCK-8 assay and it was found that downregulation of GPX3 accelerated cell proliferation at both 48 and 72 h (Fig. 2C) for both cell lines. Moreover, our flow cytometry assay showed that the ratio of cells in the G0/G1 phase was shortened in si-GPX3 transfected prostate cells with a corresponding increase of G2 phase but no significant change of S phase (Fig. 2D, E). Accordingly, G0/G1 phase cell cycle-related proteins (Cyclin D1, CDK4 and CDK6) underwent significant increase in prostate cells treated with si-GPX3, while G2 phase cell cycle-related proteins (Cyclin B1 and CDK1) were no significant difference (Fig. 2F and Additional file 6: Fig. S2B). Flow cytometry analysis also manifested that down-regulation of GPX3 restrained apoptosis of prostate cells by more than 50% (Fig. 2G, H). Meanwhile, we used 40 μM apoptosis-inducer isosilybin B (acting on the G0/G1 phase of the cell cycle. The IC_50_ of isosilybin B on cytotoxic of BPH-1 and WPMY-1 cells were 67.72 μM and 41.38 μM, respectively) for BPH-1 or 20 μM isosilybin B for WPMY-1 in combination with si-GPX3s, and the increase in apoptosis induced by isosilybin B treatment could be inhibited by the down-regulation of GPX3 (Additional file 6: Fig. S2C–E), which demonstrated that the down-regulation of GPX3 promoted cell survival by accelerating the G0/G1 phase. Furthermore, the active condition of mitochondria was determined via the detection of mitochondrial membrane potential (MMP). And the silence of GPX3 contributed to more agminated MMP [the ratio of JC-1 aggregates (orange) to JC-1 monomer (blue) increased] in prostate cells (Fig. 2I, J), indicating the initiation of apoptosis process was lessened by the more stable mitochondria. Since the release of Cytochrome C (Cyto-C) from mitochondria to cytoplasm is the committed step for concatenating mitochondrial damage to apoptosis, we performed mitochondrial and cytoplasmic compartments isolation on prostate cell lines. The knockdown of GPX3 contributed to the increase of mitochondrial Cyto-C (Cyto-C^mito^) and the decrease of cytoplasmic Cyto-C (Cyto-C^cyto^) (Fig. 2K and Additional file 6: Fig. S2F). Moreover, the release of Cyto-C is also affected by pro-apoptotic protein BAX and anti-apoptotic protein Bcl-2. When receiving apoptotic signal, caspase 9 and caspase 3 are further activated step by step to initiate apoptosis. And immunoblotting analysis showed that GPX3 silencing promoted the expression of Bcl-2 and suppressed the expression of BAX. Notably, the expression of cleaved-caspase 9 and cleaved-caspase 3 were also prominently decreased, confirming that GPX3 silencing inhibited mitochondrial-mediated apoptosis in prostatic cells (Fig. 2L and Additional file 6: Fig. S2G). The MAPK family was also altered with phosphorylated ERK1/2 (p-ERK1/2) significantly weakened but no effect on p-JNK and p-p38 (Fig. 2M and Additional file 6: Fig. S2H) in si-GPX3 treated prostate cells. In addition, to verify the feasibility of the control group, we used the untreated control (con) group to replicate with the transfection control (si-con) group and the empty plasmid (vector) group. It was showed that there was no significant difference in GPX3 protein expression level (Additional file 7: Fig. S3A, B), cell proliferation activity (Additional file 7: Fig. S3C), cell cycle (Additional file 7: Fig. S3D, E), apoptosis level (Additional file 7: Fig. S3F, G), MMP level (Additional file 7: Fig. S3H, I) and MAPK pathway protein expression level (Additional file 7: Fig. S3J, K) among the above three groups.

### Overexpression of GPX3 inhibited cell proliferation, induced G0/G1 phase arrest and triggered mitochondria-mediated apoptosis and mitochondrial dysfunction via activating ERK pathway in prostate cells

**Fig. 3:**
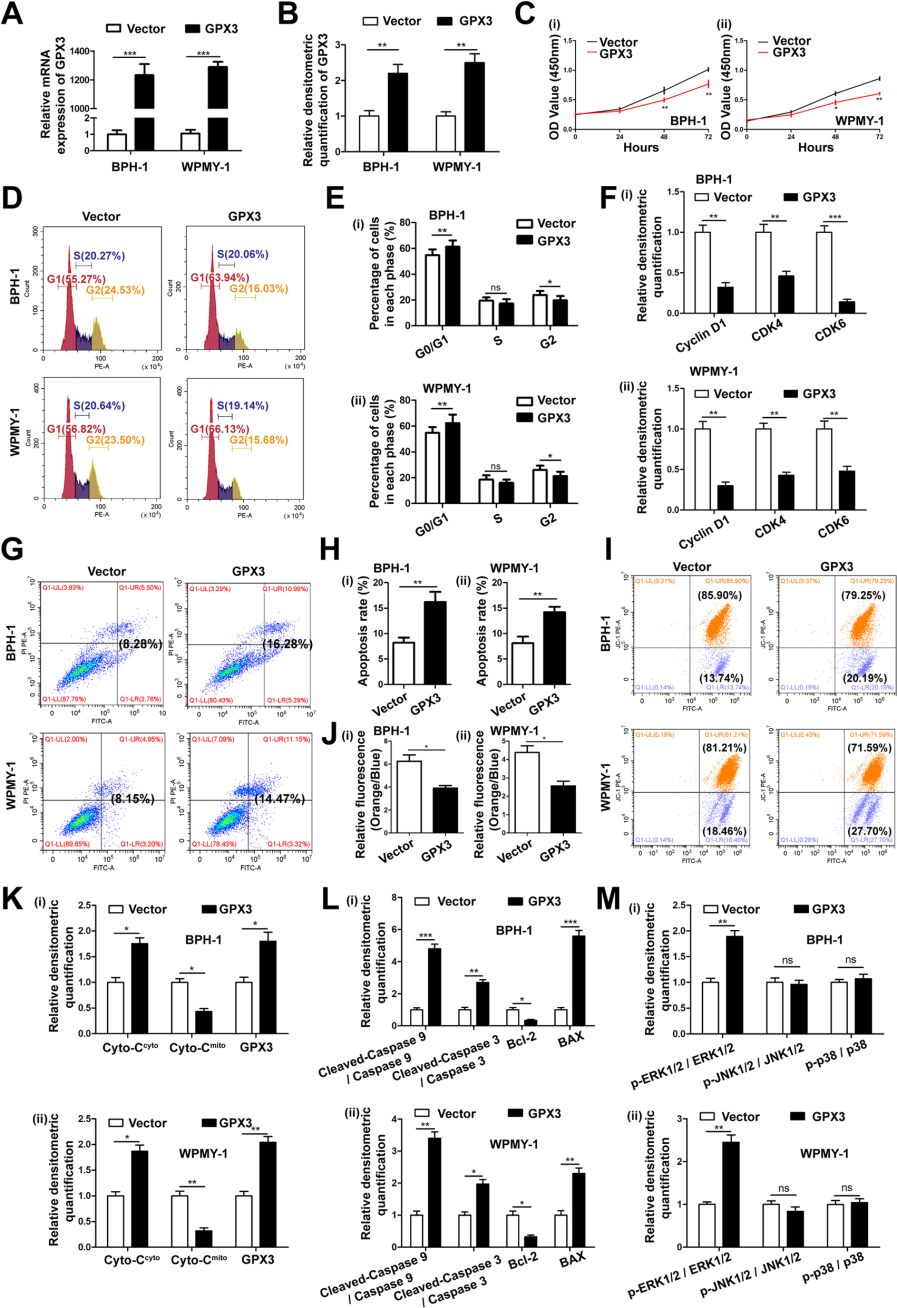
Effect of GPX3 overexpression on cell proliferation, cell cycle, apoptosis, mitochondrial membrane potential and MAPK signaling pathway in prostate cells. **A** qRT -PCR validated the efficiency of GPX3 overexpression at transcriptional level in BPH-1 and WPMY-1 cells. **B** Relative densitometric quantification of GPX3 protein in BPH-1 and WPMY-1 after overexpression of GPX3 **C** The cell viability of BPH-1 and WPMY-1 after overexpression of GPX3 at different time points by CCK-8 assay, and GPX3 overexpression inhibited cell proliferation activity. **D** Flow cytometry analysis of cell cycle. **E** Histogram showing percentage of cell populations at different stages of the cell cycle (%). **F** Relative densitometric quantification of Cyclin D1, CDK4 and CDK6 protein in BPH-1 and WPMY-1 after overexpression of GPX3. This indicated that GPX3 overexpression arrested the G0/G1 phase of prostate cells and inhibited the expression of G0/G1 checkpoint proteins. **G** Flow cytometry analysis of cell apoptosis. **H** Statistical analysis of apoptotic rate (%), and GPX3 overexpression promoted the apoptosis of prostate cells. **I** The mitochondrial membrane potential level of BPH-1 and WPMY-1 cells was examined by JC-1 staining. The scatter plot of the flow cytometry analysis shows the distribution of JC-1 aggregates (Orange) and JC-1 monomer (Blue) cell population. **J** Histogram calculated the relative ratio of Orange against Blue fluorescence, and GPX3 overexpression led to MMP dissipation in prostate cells. **K** Relative densitometric quantification of Cyto-C protein in mitochondria or cytoplasm in BPH-1 and WPMY-1 after overexpression of GPX3. **L** Relative densitometric quantification of Cleaved-Caspase 9, Cleaved-Caspase 3, Bcl-2 and BAX protein in BPH-1 and WPMY-1 after overexpression of GPX3. This suggested that GPX3 overexpression promoted the increase of mitochondria-mediated apoptosis and pro-apoptotic proteins, and decreased the anti-apoptotic protein. **M** Relative densitometric quantification of MAPK signaling pathway proteins in BPH-1 and WPMY-1 after overexpression of GPX3, and GPX3 overexpression promoted the expression of ERK1/2 protein. ^*^p < 0.05, ^**^p < 0.01, ^***^p < 0.001 and ns means no significant difference

Both the mRNA and protein levels of GPX3 were up-regulated when we transfected the plasmid into prostate cells to overexpress GPX3 for 48 h or 72 h, respectively (Fig. 3A, B and Additional file 8: Fig. S4A). The CCK-8 assay manifested that GPX3 restrained cell growth (Fig. 3C). Furthermore, the flow cytometry assay observed that the proportion of cells in the G0/G1 phase was elevated with a decrease of G2 phase and no significant change of S phase (Fig. 3D, E), accompanied by a decreased expression of cycle-related proteins (Cyclin D1, CDK4 and CDK6) in GPX3 overexpressed prostate cells (Fig. 3F and Additional file 8: Fig. S4B). Meanwhile, overexpression of GPX3 facilitated cell apoptosis (Fig. 3G, H) and resulted in dissipative MMP [the ratio of JC-1 aggregates (orange) to JC-1 monomer (blue) decreased] in prostate cells (Fig. 3I, J). Moreover, it was observed GPX3 overexpression enhanced the translocation of Cyto-C from mitochondria to cytoplasm (Fig. 3K and Additional file 8: Fig. S4C) and led to the up-regulation of BAX, cleaved-caspase 9 and cleaved-caspase 3 with the expression of Bcl-2 suppressed (Fig. 3L and Additional file 8: Fig. S4D). Additionally, the upregulation of GPX3 significantly strengthened the phosphorylation of ERK1/2 in the MAPK family (Fig. 3M and Additional file 8: Fig. S4E).

### U0126 reversed the effects of GPX3 overexpression on cell proliferation activity, cell cycle arrest and apoptosis

**Fig. 4:**
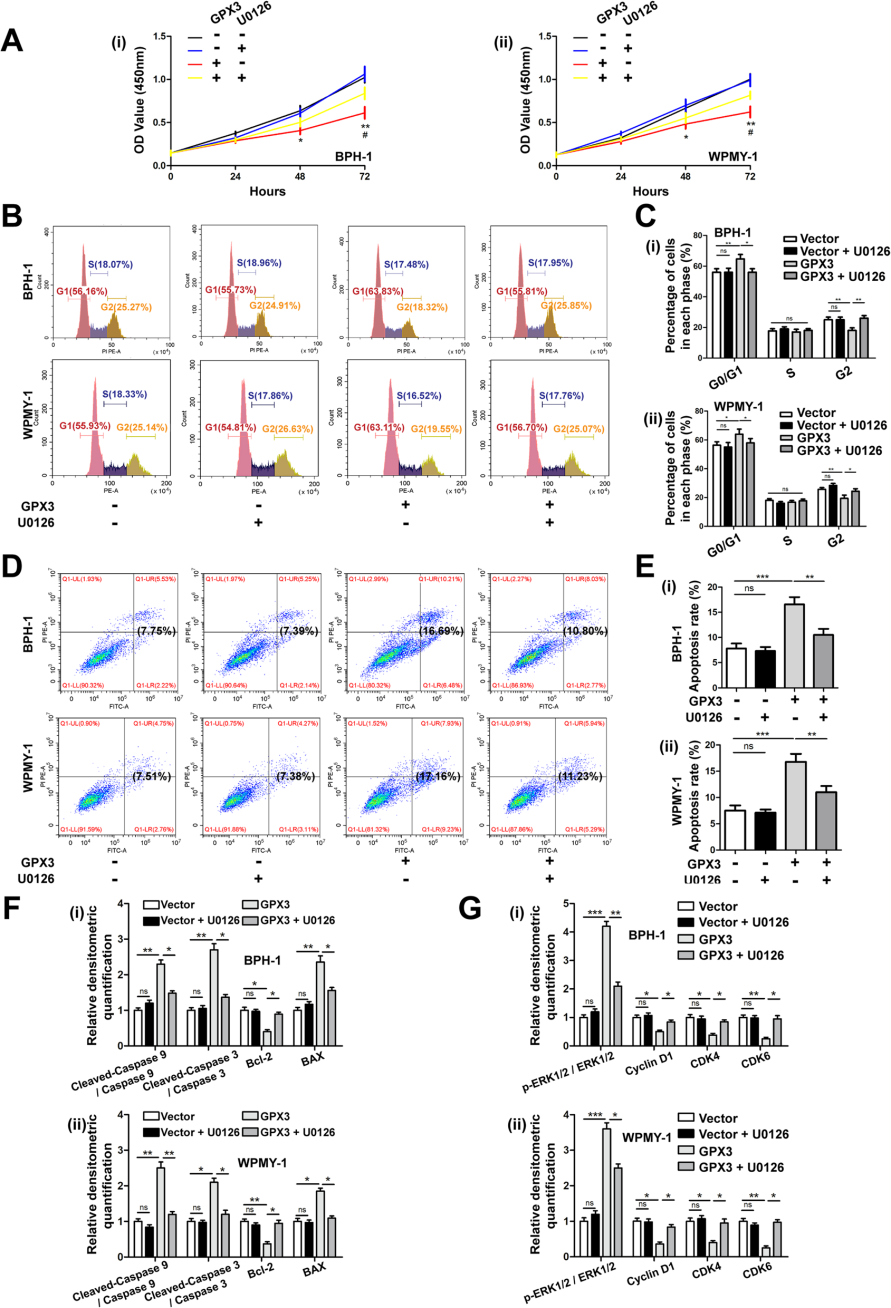
Effect of U0126 on proliferation activity, cell cycle and apoptosis of prostate cells after GPX3 overexpression **A** BPH-1 and WPMY -1 cells were pre-treated by U0126 at 10 μM for 24 h and treated by GPX3 overexpression plasmid for 48 h, comparing with vector cells. Cell proliferation of the BPH-1 and WPMY-1 cells was analyzed by CCK-8 assay, and U0126 reversed attenuated cell proliferation viability caused by GPX3 overexpression. ^*^: Vector vs. GPX3; ^#^: GPX3 vs. GPX3 + U0126. **B** Flow cytometry analysis of cell cycle. **C** Histogram showing percentage of cell populations at different stages of the cell cycle (%), and U0126 reversed G0/G1 phase arrest caused by GPX3 overexpression. **D** Flow cytometry analysis of cell apoptosis. **E** Statistical analysis of apoptotic rate (%), and U0126 reversed the increased apoptosis caused by GPX3 overexpression. **F** Relative densitometric quantification of Cleaved-Caspase 9, Cleaved-Caspase 3, Bcl-2 and BAX protein in BPH-1 and WPMY-1, and U0126 reversed the increased pro-apoptotic proteins and decreased anti-apoptotic protein caused by GPX3 overexpression. **G** Relative densitometric quantification of phosphorylated and total ERK1/2 as well as cell cycle related proteins (Cyclin D1, CDK4 and CDK6), and U0126 recovered the expression of G0/G1 phase checkpoint proteins and p-ERK1/2 changed by GPX3 overexpression. ^*/#^*p* < 0.05, ^**^p < 0.01, ^***^p < 0.001 and ns means no significant difference

The prostate cells were treated with U0126 (MEK1/2 pathway inhibitor) and GPX3 overexpressed plasmid alone or together. As expected, U0126 significantly reversed attenuated cell proliferation viability (Fig. 4A), G0/G1 phase arrest (Fig. 4B, C) and increased apoptosis (Fig. 4D, E) caused by GPX3 overexpression. Furthermore, U0126 recovered the expression of apoptosis-related proteins (BAX, Bcl-2, cleaved-caspase 9 and cleaved-caspase 3) (Fig. 4F and Additional file 8: Fig. S4F), cycle-related proteins (Cyclin D1, CDK4 and CDK6) and p-ERK1/2 changed by GPX3 overexpression in BPH-1 and WPMY-1 cell lines to a certain extent (Fig. 4G and Additional file 8: Fig. S4G).

### GPX3 silencing induced ferroptosis in prostate cells with the participation of GPX4

**Fig. 5:**
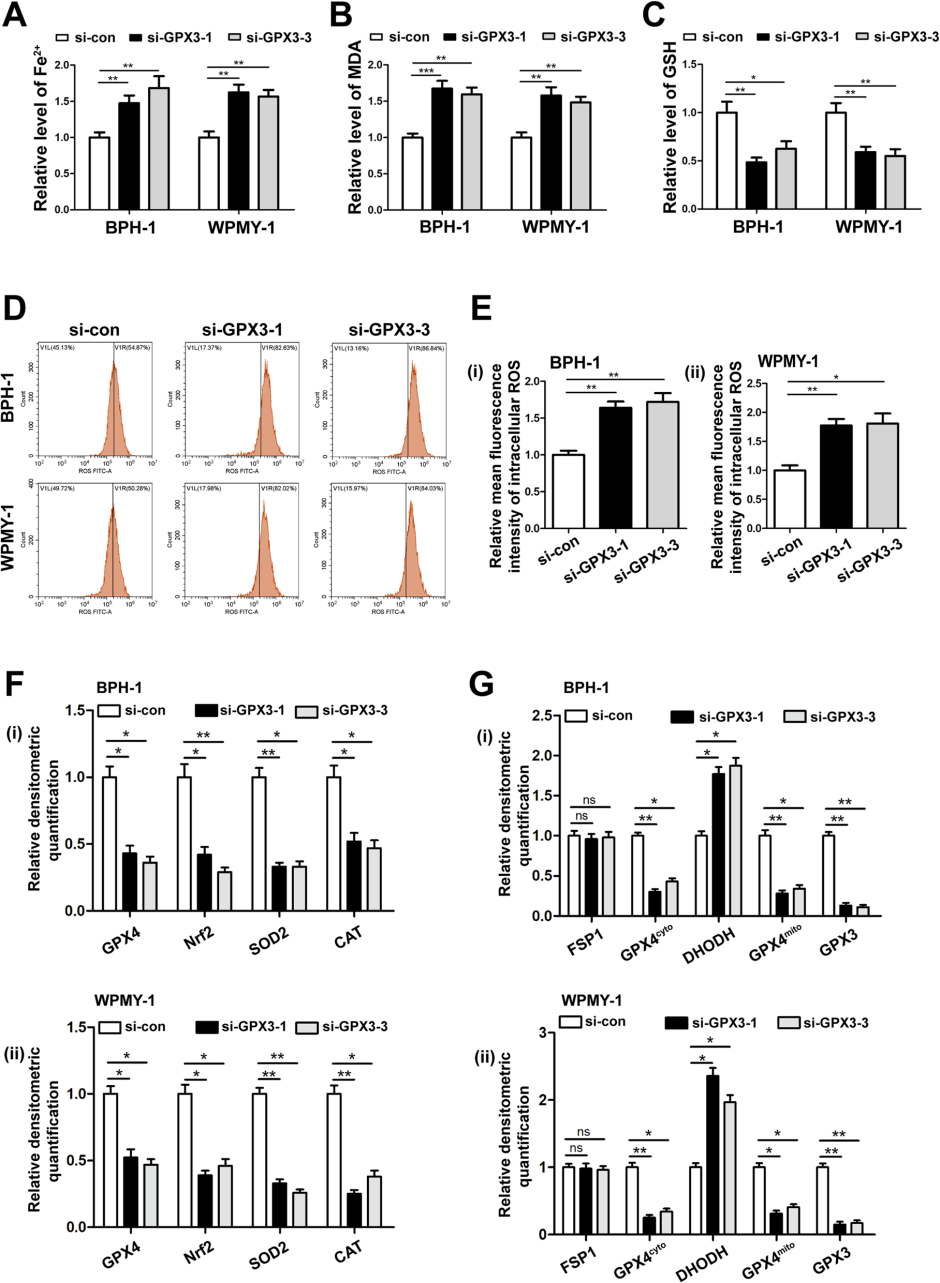
Effect of GPX3 knockdown on frroptosis and ROS in prostate cells. The content of iron (**A**), MDA (**B**) and GSH (**C**) in BPH-1 and WPMY-1 cells were detected by colorimetric assay kit, and results indicated that GPX3 silencing up-regulated the level of ferroptosis.** D** DCFH-DA fluorescent probe was used to analyze the accumulation of ROS by GPX3 knockdown in BPH-1 and WPMY-1 cells. **E** Statistical analysis of mean fluorescence intensity (MFI) of DCFH-DA, and GPX3 silencing promoted ROS accumulation in prostate cells. **F** Relative densitometric quantification of proteins in relation to ferroptosis (GPX4 and Nrf2) and OS (SOD2 and CAT) in BPH-1 and WPMY-1 after knockdown of GPX3, and knockdown of GPX3 amplified the ferroptosis and ROS levels in prostate cells. **G** Relative densitometric quantification of GPX4, FSP1 and DHODH in mitochondria or cytoplasm were detected after knockdown of GPX3, and the results showed that GPX3 knockdown regulated ferroptosis mainly with the participation of GPX4. ^*^p < 0.05, ^**^p < 0.01, ^***^p < 0.001 and ns means no significant difference

In order to explore the effect of GPX3 on ferroptosis in prostate cells, we detected the intracellular iron, lipid peroxidation product MDA and substrate GSH after GPX3 knockdown. It was observed that GPX3 silencing increased the content of iron (Fig. 5A) and MDA (Fig. 5B) while decreased the level of GSH (Fig. 5C) in prostate cells. At the same time, the level of intracellular ROS increased (Fig. 5D, E). Consistently, immunoblotting analysis indicated that si-GPX3 induced the down-regulation of nuclear factor erythroid 2-related factor-2 (Nrf2), glutathione peroxidase 4 (GPX4) and OS markers [superoxide dismutase 2 (SOD2) and catalase (CAT)] (Fig. 5F and Additional file 9: Fig. S5A). All these changes suggested GPX3 silence amplified ferroptosis. Since mitochondria is the important part of the regulation of ferroptosis, we performed mitochondrial and cytoplasmic compartments isolation on prostate cell lines. The knockdown of GPX3 contributed to the decrease of mitochondrial GPX4 (GPX4^mito^) and cytoplasmic GPX4 (GPX4^cyto^) and an increase of dihydroorotate dehydrogenase (DHODH, anti-ferroptosis system exists only in mitochondria) with no significant change of cytoplasmic ferroptosis suppressor protein 1 (FSP1, anti-ferroptosis system exists only in cytoplasm) (Fig. 5G and Additional file 9: Fig. S5B), indicating silenced GPX3 enhanced ferroptosis both in the cytoplasm and mitochondria with the involvement of dysfunctional GPX4^mito^ and GPX4^cyto^, and was accompanied by upregulation of mitochondrial DHODH responsiveness.

### Overexpression of GPX3 reversed the RSL3 induced ferroptosis

**Fig. 6:**
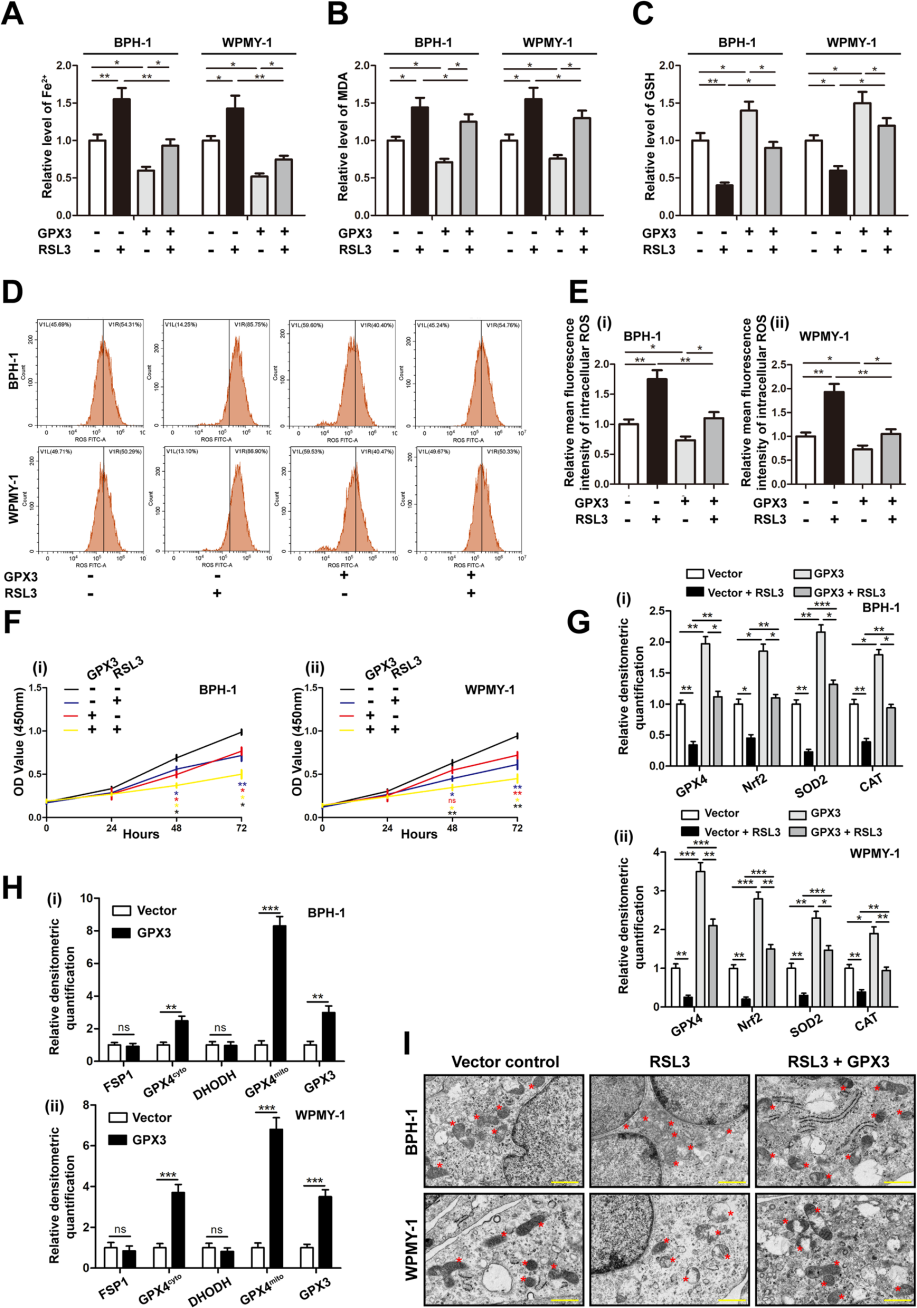
Effect of GPX3 overexpression on RSL3-mediated ferroptosis in prostate cells. BPH-1 and WPMY -1 cells were pre-treated by RSL3 at 0.5 μM for 24 h and treated by GPX3 overexpression plasmid for 48 h. The content of iron **A**, MDA (**B**) and GSH (**C**) in BPH-1 and WPMY-1 cells were detected by colorimetric assay kit, and GPX3 overexpression reversed RSL3-induced ferroptosis. **D** DCFH-DA fluorescent probe was used to analyze the accumulation of ROS in BPH-1 and WPMY-1 cells. **E** Statistical analysis of mean fluorescence intensity (MFI) of DCFH-DA, and GPX3 overexpression reversed RSL3-induced ROS accumulation. **F** Cell proliferation of the BPH-1 and WPMY-1 cells was analyzed by CCK-8 assay. Both RSL3 treatment and GPX3 overexpression could attenuate the proliferation ability of prostate cells, and the two have the superposition effect. ^*^: Vector vs. Vector + RSL3; ^*^: Vector vs. Vector + GPX3; ^*^: Vector + RSL3 vs. RSL3 + GPX3; ^*^: GPX3 vs. RSL3 + GPX3. **G** Relative densitometric quantification of proteins in relation to ferroptosis (GPX4 and Nrf2) and OS (SOD2 and CAT) in BPH-1 and WPMY-1 cells, and GPX3 overexpression reversed the inhibited ferroptosis system and antioxidant enzymes caused by RSL3 treatment. **H** Relative densitometric quantification of GPX4, FSP1 and DHODH in mitochondria or cytoplasm was detected after overexpression of GPX3, and the results showed that GPX3 overexpression regulated ferroptosis mainly with the participation of GPX4. **I** Control vector or GPX3 plasmid were transfected into BPH-1 and WPMY-1 cells and treated with RSL3 (0.5 μM) for 24 h. Transmission electron microscopy was used to examine the typical changes of ferroptotic cells, and GPX3 overexpression reversed the mitochondrial changes of ferroptosis caused by RSL3 treatment. Scale bars: 500 nm. Representative photographs were showed. Red stars represented mitochondrion. ^*^p < 0.05, ^**^p < 0.01, ^***^p < 0.001 and ns means no significant difference

To further validate GPX3 mediated ferroptosis with the participation of GPX4, prostate cells were pretreated with RSL3 (GPX4 inhibitor/ferroptosis agonist) and transfected with GPX3 overexpressed plasmid alone or together. The viability of BPH-1 and WPMY-1 cells treated with RSL3 was detected by CCK-8 assay, and the half-maximal inhibitory concentration (IC_50_) of RSL3 was 0.71 μM and 0.75 μM, respectively (Additional file 9: Fig. S5C). Based on the IC_50_, 0.5 μM dosage of RSL3 was chosen. As shown in Fig. 6A–E, RSL3 treatment could induce the increase of iron content, MDA, intracellular ROS and the decrease of GSH in prostate cells, while GPX3 overexpression up-regulated the level of GSH, and down-regulated the content of iron, MDA and intracellular ROS in a RSL3-independent manner. In addition, in the detection of cell proliferation activity, both RSL3 treatment and GPX3 overexpression could attenuate the proliferation ability of prostate cells, and which was aggravated by RSL3 treatment and GPX3 overexpression combined (Fig. 6F). Meanwhile, ferroptosis-related proteins (GPX4 and Nrf2) and OS markers (SOD2 and CAT) were accordingly changed (Fig. 6G and Additional file 9: Fig. S5D). Moreover, we performed mitochondrial and cytoplasmic compartment isolation on GPX3 overexpressed BPH-1 and WPMY-1 cell lines. It was observed GPX3 overexpression enhanced the expression of GPX4^cyto^ and GPX4^mito^, but FSP1 and DHODH did not change significantly (Fig. 6H and Additional file 9: Fig. S5E). More importantly, transmission electron microscopy (TEM) assays confirmed that RSL3 treatment substantially shanked mitochondria and decreased the number of ridges, whereas GPX3 plasmid largely abolished the typical changes of ferroptotic cells (Fig. 6I). Overall, these data indicated that GPX3 overexpressed exerted anti-ferroptosis effects both in the cytoplasm and mitochondria with the participation of GPX4.

### GPX3 regulated ferroptosis in an autophagy-related manner

**Fig. 7:**
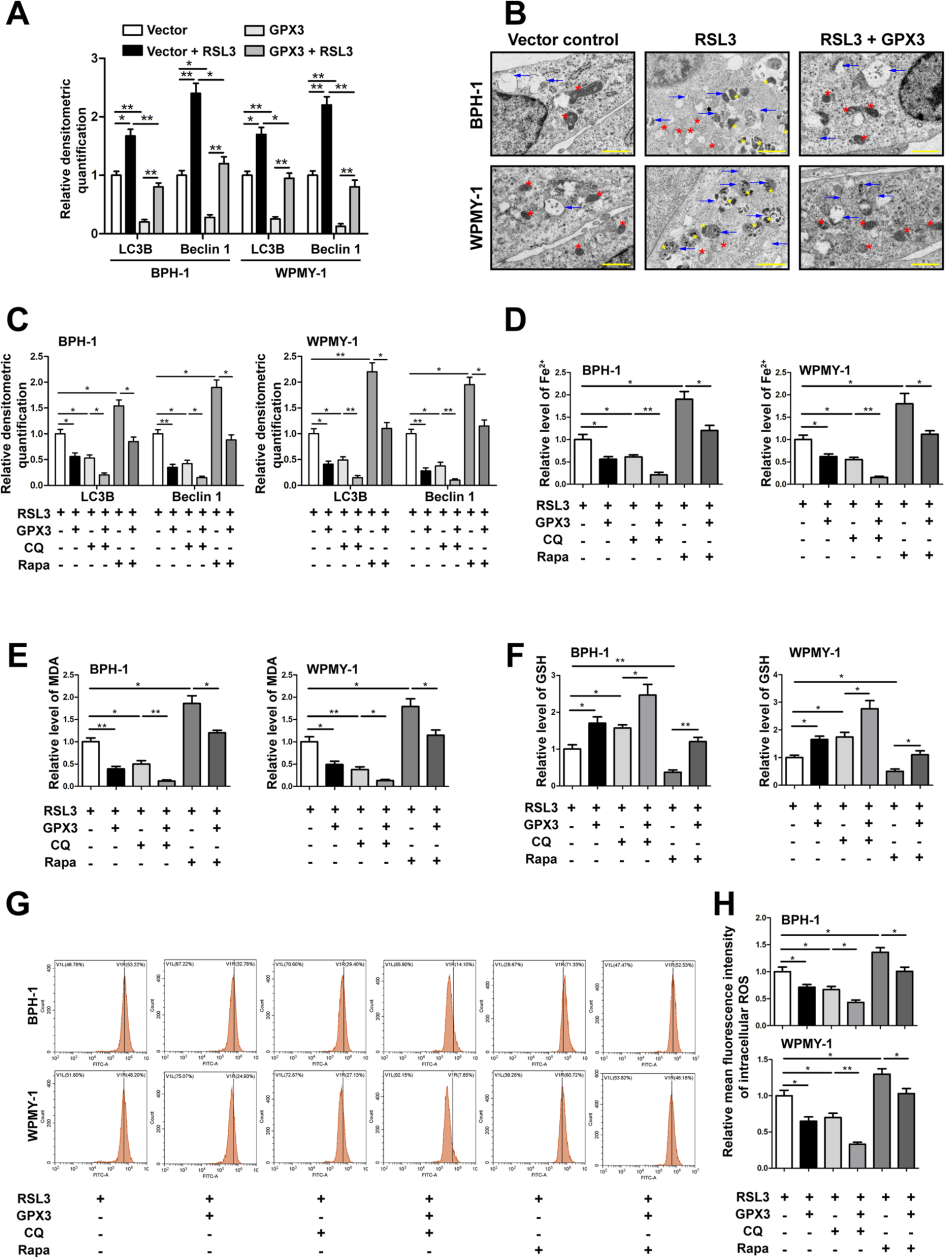
GPX3 regulated autophagy-related ferroptosis **A** Relative densitometric quantification of proteins in relation to autophagy (LC3B and Beclin1) in BPH-1 and WPMY-1 cells by GPX3 overexpression or RSL3 treatment.** B** Control vector or GPX3 plasmid were transfected into BPH-1 and WPMY-1 cells and treated with RSL3 (0.5 μM) for 24 h. Transmission electron microscopy was used to examine the typical changes of autophagic vacuoles. Scale bars: 500 nm. Representative photographs were showed. Blue arrows represented autophagic vesicles. Red stars represented mitochondrion. Yellow stars represented lysosomes. The results showed that GPX3 overexpression reversed the increased autophagy level caused by RSL3 treatment. **C** BPH-1 and WPMY-1 cells were transfected with control vector or GPX3 plasmid. Subsequently, transfected cells were treated with RSL3 (0.5 μM) for 24 h, with or without the rapamycin (Rapa, 2 nM) and chloroquine (CQ, 20 μM) for 24 h. Relative densitometric quantification of proteins in relation to autophagy (LC3B and Beclin1) in BPH-1 and WPMY-1 cells, which confirmed the activation of autophagy by Rapa and the inhibition of autophagy by CQ. The content of iron **D**, MDA (**E**) and GSH **F** in BPH-1 and WPMY-1 cells were detected by colorimetric assay kit. **G** DCFH-DA fluorescent probe was used to analyze the accumulation of ROS in BPH-1 and WPMY-1 cells. **H** Statistical analysis of mean fluorescence intensity (MFI) of DCFH-DA. The results showed that autophagy was positively correlated with ferroptosis in prostate cells, and GPX3 overexpression had both anti-autophagy and anti-autophagy-related ferroptosis effects. ^*^p < 0.05, ^**^p < 0.01

We also examined the levels of Beclin1 and LC3B, which are the initial protein and structural components of autophagosomes, respectively. Immunoblotting analysis showed that RSL3 could significantly up-regulate the expression of LC3B and Beclin1, while GPX3 overexpression could down-regulate the expression of LC3B and Beclin1 (Fig. 7A and Additional file 10: Fig. S6A). Meanwhile, TEM analysis further showed that RSL3 increased the autophagosome formation in prostate cells, which could be reversed by GPX3 overexpression (Fig. 7B). In order to further elucidate the relationship between ferroptosis and autophagy in prostate cells, we combined 2 nM Rapa (autophagy agonist. The IC_50_ of Rapa on cytotoxic of BPH-1 and WPMY-1 cells were 2.67 nM and 3.24 nM, respectively) with 20 μM CQ (autophagy inhibitor. The IC_50_ of CQ on cytotoxic of BPH-1 and WPMY-1 cells were 28.52 μM and 31.04 μM, respectively) (Additional file 10: Fig. S6B, C). Western blot indicated that under the premise of RSL3 pretreatment, GPX3 overexpression aggravated the down-regulation of LC3B and Beclin1 expression induced by CQ, and weakened the up-regulation of LC3B and Beclin1 expression triggered by Rapa (Fig. 7C and Additional file 10: Fig. S6D). Moreover, there were superimposed effects on CQ‐induced decrease of iron content, MDA, intracellular ROS and the increase of GSH, compared to prostate cells treated with GPX3 overexpression alone (Fig. 7D–H). Conversely, Rapa increased the levels of iron content, MDA and intracellular ROS, and decreased the GSH in prostate cells, which could be reversed by GPX3 overexpression (Fig. 7D–H). The above results indicated that GPX3 in prostate cells could inhibit autophagy-related ferroptosis by antagonizing autophagy.

### GPX3 antagonized autophagy-related ferroptosis and activated ERK1/2 with the involvement of adenosine monophosphate-activated protein kinase (AMPK) pathway

**Fig. 8:**
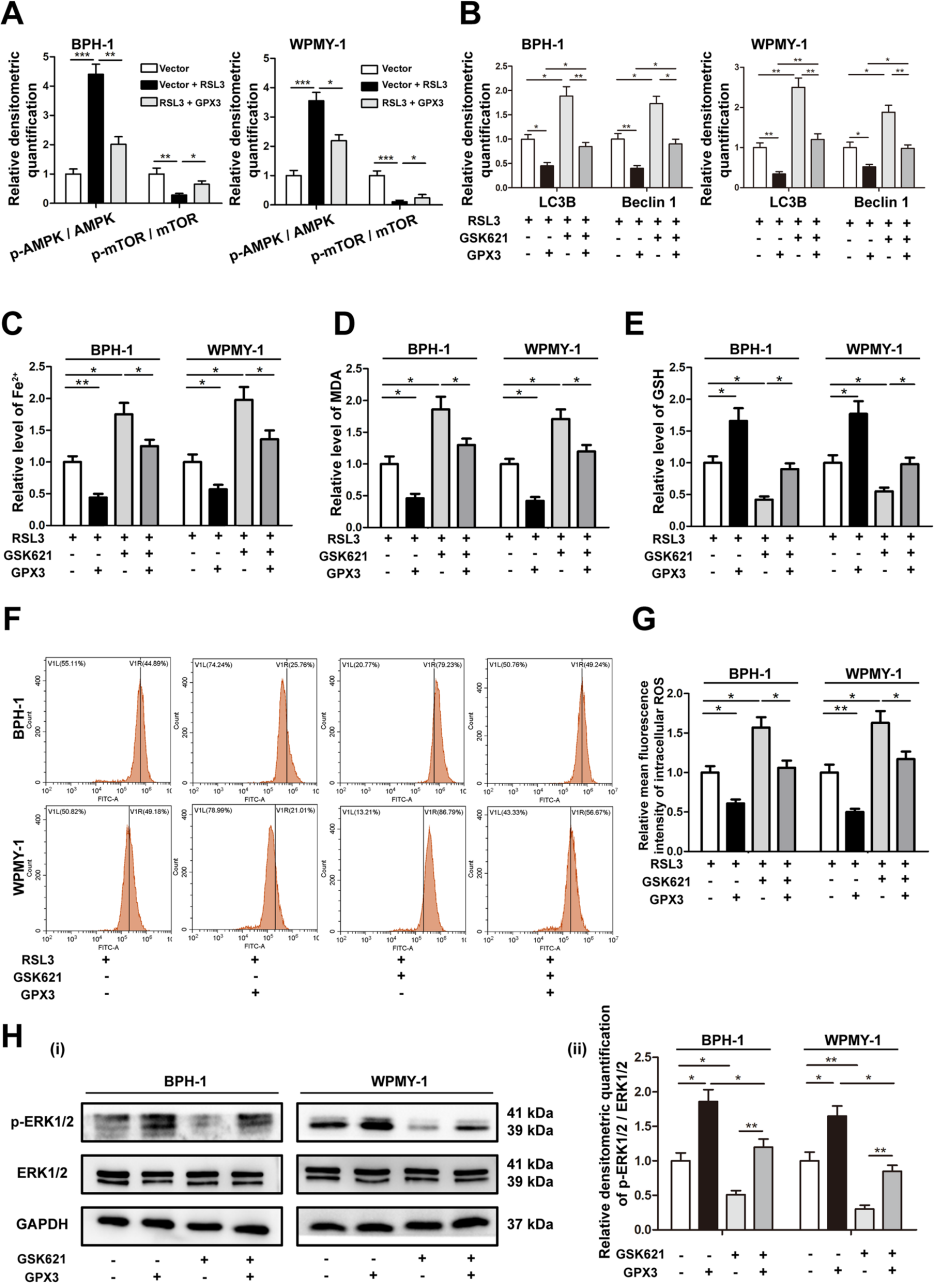
GPX3 antagonized autophagy-related ferroptosis and activated ERK1/2 with the involvement of AMPK **A** Relative densitometric quantification of proteins (AMPK, p-AMPK, mTOR and p-mTOR) in BPH-1 and WPMY-1 cells treated with RSL3 (0.5 μM) or GPX3 plasmid, and GPX3 overexpression reversed the up-regulation of p-AMPK and down-regulation of p-mTOR caused by RSL3 treatment. **B** Relative densitometric quantification of proteins (LC3B and Beclin1) in BPH-1 and WPMY-1 cells treated with GPX3 plasmid or GSK621 (20 μM), and GPX3 overexpression reversed the up-regulation of autophagy caused by GSK621 treatment. The content of iron (**C**), MDA (**D**) and GSH (**E**) in BPH-1 and WPMY-1 cells treated with GPX3 plasmid or GSK621 (20 μM) were detected by colorimetric assay kit. **F** DCFH-DA fluorescent probe was used to analyze the accumulation of ROS in BPH-1 and WPMY-1 cells treated with GPX3 plasmid or GSK621 (20 μM). **G** Statistical analysis of mean fluorescence intensity (MFI) of DCFH-DA. The results indicated that GPX3 overexpression reversed the up-regulation of autophagy-related ferroptosis caused by GSK621 treatment. **H** Immunoblot assay (i) and relative densitometric quantification (ii) of ERK1/2 and p-ERK1/2 in BPH-1 and WPMY-1 cells treated with 20 μM GSK621 or GPX3 plasmid showed that GPX3 regulated ERK1/2 signaling with the involvement of AMPK pathway. GAPDH is used as loading control. ^*^p < 0.05, ^**^p < 0.01, ^***^p < 0.001

AMPK is a positive regulator of autophagy via suppressing mammalian target of rapamycin (mTOR) [37]. To investigate the role of AMPK/mTOR pathway in GPX3-mediated autophagy inhibition, we initially detected the activation levels of AMPK and mTOR and it was found that GPX3 overexpression inhibited the increase of AMPK phosphorylation and the decrease of mTOR phosphorylation triggered by RSL3 treatment (Fig. 8A and Additional file 10: Fig. S6E). We further used 20 μM GSK621 (AMPK agonist, the IC_50_ of GSK621 on cytotoxic of BPH-1 and WPMY-1 cells were 23.21 μM and 25.33 μM, respectively) (Additional file 10: Fig. S6F) pretreatment to determine whether AMPK was associated with GPX3-mediated autophagy. And the protein expression of p‐AMPK was significantly up-regulated in GSK621‐treated cells (Additional file 10: Fig. S6G). As shown in Fig. 8B and Additional file 10: Fig. S6H, GSK621 up-regulated the expression of LC3B and Beclin1, which could be partially rescued by GPX3 overexpression. In addition, GSK621 also mediated the upregulation of intracellular iron content, MDA and ROS, and the depletion of GSH, which were also reversed to some extent by GPX3 overexpression (Fig. 8C–G). Data suggested that GPX3 mediated autophagy-related ferroptosis of prostate cells with the involvement of AMPK/mTOR pathway. Moreover, AMPK acts as an upstream negative regulator of ERK1/2. Indeed, we observed GSK621 inhibited the expression of p-ERK1/2 protein, which could be reversed by GPX3 overexpression (Fig. 8H). It was suggested that GPX3 regulated ERK1/2 signaling with the involvement of AMPK pathway.

### The level of apoptosis in hyperplastic prostate decreased, while OS, ferroptosis and autophagy increased

**Fig. 9:**
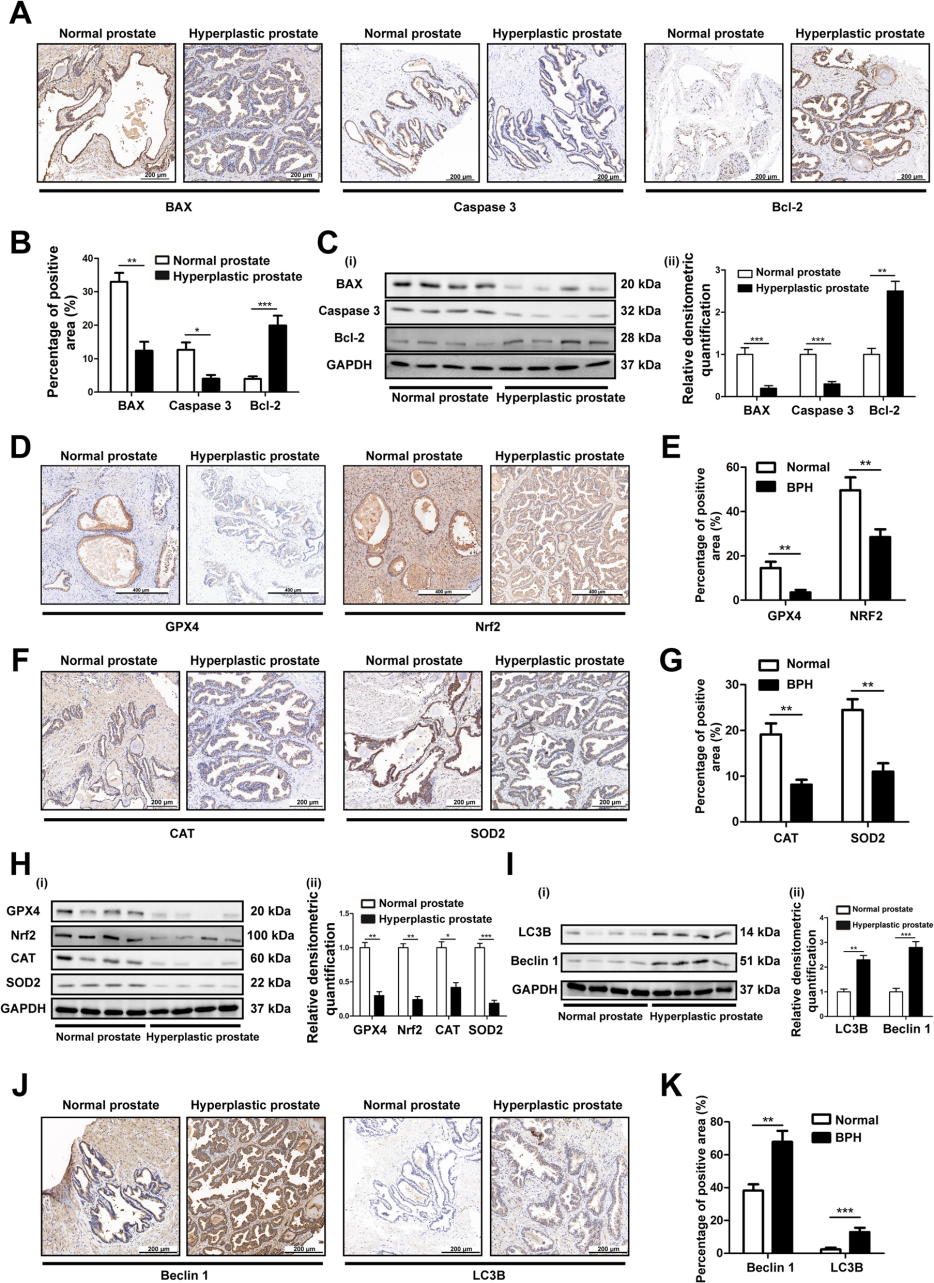
The levels of apoptosis, OS, ferroptosis and autophagy in normal and hyperplastic prostates **A** Representative IHC images of BAX, Caspase 3 and Bcl-2 in human prostate tissues. The scale bars are 200 μm. **B** The quantitative analysis of BAX, Caspase 3 and Bcl-2 expression in human prostate tissues. **C** Immunoblot assay (i) and relative densitometric quantification (ii) revealed the protein expression of BAX, Caspase 3 and Bcl-2 in normal prostate tissues (n = 4) and BPH tissues (n = 4). The results showed that there was a lower level of apoptosis in human hyperplastic prostate. **D** Representative IHC images of GPX4 and Nrf2 in human prostate tissues. The scale bars are 400 μm. **E** The quantitative analysis of GPX4 and Nrf2 expression in human prostate tissues. **F** Representative IHC images of CAT and SOD2 in human prostate tissues. The scale bars are 200 μm. **G** The quantitative analysis of CAT and SOD2 expression in human prostate tissues. **H** Immunoblot assay (i) and relative densitometric quantification (ii) revealed the protein expression of GPX4, Nrf2, CAT and SOD2 in normal prostate tissues (n = 4) and BPH tissues (n = 4). The results showed that there were higher levels of ferroptosis and OS in human hyperplastic prostate. **I** Immunoblot assay (i) and relative densitometric quantification (ii) revealed the protein expression of LC3B and Beclin1 in normal prostate tissues (n = 4) and BPH tissues (n = 4). **J** Representative IHC images of Beclin1 and LC3B in human prostate tissues. The scale bars are 200 μm. **K** The quantitative analysis of Beclin1 and LC3B expression in human prostate tissues. The results showed that there was a higher level of autophagy in human hyperplastic prostate. GAPDH is used as loading control. ^*^p < 0.05, ^**^p < 0.01, ^***^p < 0.001

We further verified the expression of BAX, caspase 3 and Bcl-2 in tissue samples by IHC staining. The results revealed the expression of BAX and caspase 3 in the hyperplastic prostate tissues were significantly lower than that in normal tissues, while Bcl-2 was the opposite (Fig. 9A, B), which was consistent with the results of Western blotting (Fig. 9C). Moreover, to compare the levels of ferroptosis markers (GPX4 and Nrf2) and OS in normal and hyperplastic prostates, we performed IHC staining (Fig. 9D–G) and Western blotting (Fig. 9H) of GPX4, Nrf2 and antioxidant enzymes (CAT and SOD2) in normal and hyperplastic prostate tissues, and found that the expression of them declined in hyperplastic prostate tissues. Meanwhile, we verified the expression of autophagy-related factors (Beclin1 and LC3B) in tissue samples by Western blotting (Fig. 9I) and IHC staining (Fig. 9J, K). The results revealed that the expression of Beclin1 and LC3B in the hyperplastic prostate tissues were significantly higher than that in normal tissues. The above results indicated that the down-regulation of GPX3 (Fig. 1A–F) in hyperplastic prostate is accompanied by a low level of apoptosis and a high level of OS, ferroptosis and autophagy.

### GPX3 inhibited BPH progress and cell proliferation, induced cell apoptosis and cell cycle arrest while antagonized ferroptosis and autophagy in vivo

**Fig. 10:**
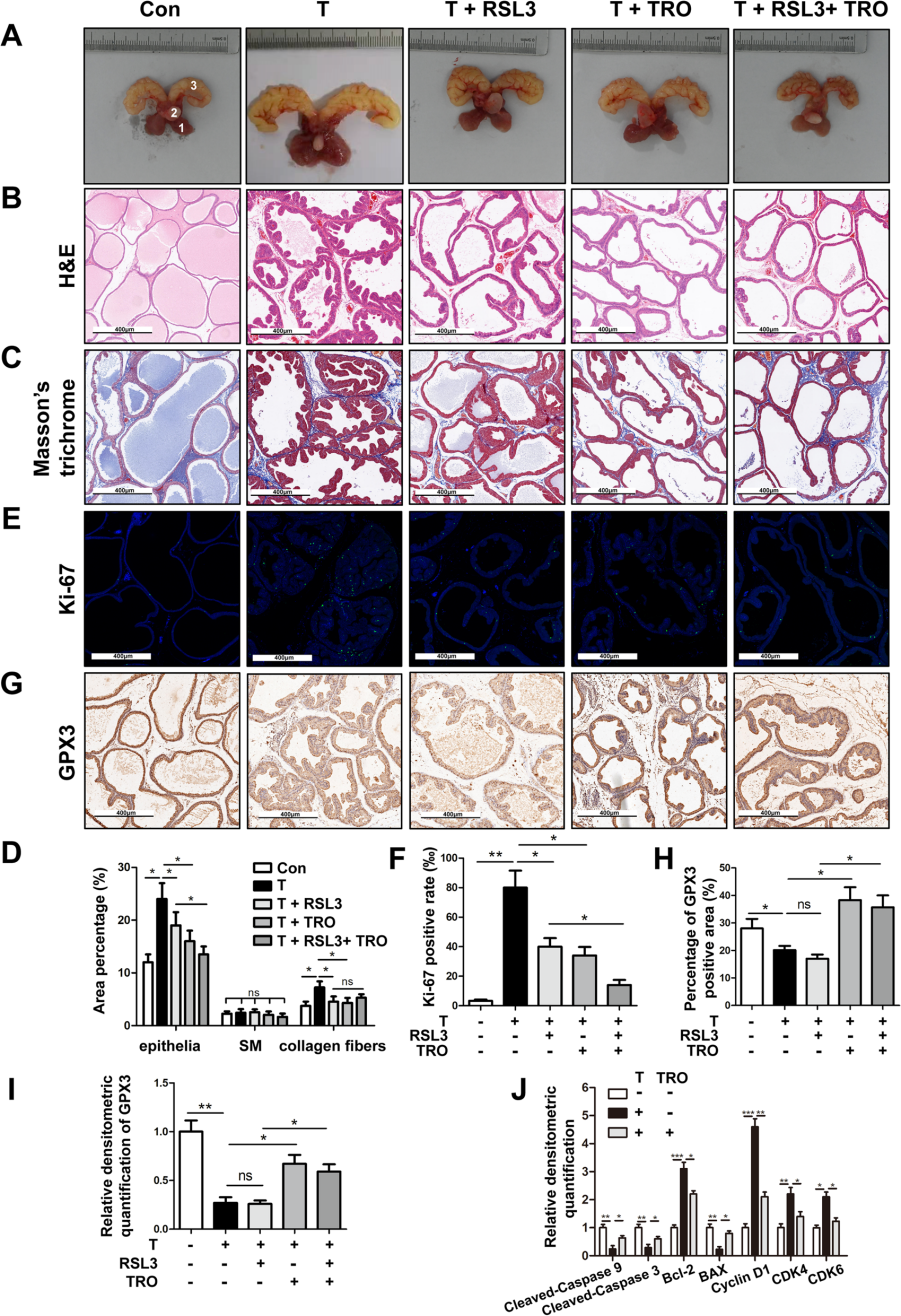
Effect of GPX3 on cell proliferation, cell cycle and apoptosis of prostate in vivo. **A** The rat urogenital tissues from Con, testosterone (T), T + RAS-selective lethal 3 (RSL3), T + troglitazone (TRO) and T + RSL3 + TRO-treated rats. (1) ventral prostate, (2) bladder and (3) seminal vesicle. **B** Representative H&E staining of rat prostates for each treatment group. Scale bars: 400 μm. **C** Masson’s trichrome staining of rat prostates for each treatment group; prostate epithelial cells were stained auburn, smooth muscle (SM) cells were stained red, and collagen fibers were stained blue. Scale bars: 400 μm. **D** Quantification of Masson’s trichrome staining. **E** The Ki-67 staining for the prostates from each group rats, respectively. DAPI (blue) and fluorescence-labeled images (green) are merged. Scale bars: 400 μm. **F** The bar graph for Ki-67-positive rate (%). The results showed the proliferative activity of prostate in T-BPH rats was significantly enhanced and could be inhibited by RSL3 or TRO. **G** Representative IHC images of GPX3 in each treatment group. Scale bars: 400 μm. **H** The quantitative analysis of GPX3 expression in each group rat prostates. **I** Relative densitometric quantification of GPX3 in prostate of each treatment group. The results indicated that TRO successfully promoted the expression of GPX3 in rat prostate. **J** Relative densitometric quantification of Cleaved-Caspase 9, Cleaved-Caspase 3, Bcl-2, BAX, Cyclin D1, CDK4 and CDK6 in prostate of Con, T, and T + TRO-treatment group, and the decrease of apoptosis and the acceleration of G0/G1 phase in the prostate of T-BPH rats could be inhibited by GPX3 upregulation in vivo. ^*^p < 0.05, ^**^p < 0.01, ^***^p < 0.001 and ns means no significant difference

Finally, we translated our in vitro study into in vivo experiment to further verify the function of GPX3. Rats supplemented with 4 week testosterone (T) showed significant increase in ventral prostate weight and prostate index (prostate weight/body weight) (Fig. 10A and (Table 1). In addition, compared with T group, RSL3 treatment significantly reduced the body weight (*p* < 0.01), ventral prostate weight (*p* < 0.001) and prostate index (*p* < 0.001). Furthermore, TRO (GPX3 agonist) treatment decreased the ventral prostate weight (*p* < 0.001) and prostate index (*p* < 0.001) while increased body weight (*p* < 0.01) when compared with T group. Moreover, compared with T + RSL3 group, T + RSL3 + TRO treatment had no significant effect on ventral prostate weight (*p* > 0.05), but decreased prostate index (*p* < 0.05) and increased body weight (* p* < 0.001) (Table 1) In the prostate of T-BPH rats, the epithelium component was relatively increased. Nevertheless, RSL3, TRO and RSL3 + TRO effectively restrained the progression of BPH induced by T. The shrunk glands were lined with a single layer of columnar epithelium to low cuboidal cells, along with slight edema, which was most significant in the T + RSL3 + TRO group (Fig. 10B**)**. Masson’s trichrome staining further indicated that the hyperplasia of the prostate occurred primarily in epithelium (*p* < 0.05) and secondarily in collagen fibers (*p* < 0.05), while smooth muscle (SM) had no difference in BPH rats. Moreover, when the T-BPH rat model treated with RSL3 or TRO, the components of epithelia and collagen fibers were relatively reduced, and the differences were statistically significant (*p* < 0.05). When compared with T + RSL3 group, T + RSL3 + TRO group only reduced the epithelial components (*p* < 0.05) with no effect on the stromal components (Fig. 10C, D). Furthermore, in order to quantify the proliferation level of rat prostates, we detected the nuclear antigen Ki-67, which is positively correlated with cell proliferation activity, and immunofluorescence staining of the rat prostate tissues showed that the positive rate of Ki-67 was significantly increased in BPH rats (*p* < 0.01) and was inhibited by RSL3 or TRO treatment *(p* < 0.05). When RSL3 and TRO treated simultaneously, the inhibition effect on the positive rate of Ki-67 was more significant than that of T + RSL3 group (*p* < 0.05) (Fig. 10E, F). Moreover IHC staining showed that the expression of GPX3 decreased in T-BPH rats (*p* < 0.05), and TRO treatment significantly upregulated the expression of GPX3 (*p* < 0.05) but there was no significant change with RSL3 treatment (*p* > 0.05) (Fig. 10G, H). The Western blot results were consistent to IHC (Fig. 10I, Additional file 11: Fig. S7A). Meanwhile, Western blot manifested that apoptosis-related proteins (BAX, cleaved-caspase 9 and cleaved-caspase 3) were down-regulated, and anti-apoptosis protein Bcl-2 and cycle-associated proteins (Cyclin D1, CDK4 and CDK6) were up-regulated in T-BPH rats, all of which were reversed by TRO intervention (Fig. 10J and Additional file 11: Fig. S7B). The above results indicated that the T-BPH rat model was successfully established, and the prostate of T-BPH rats showed accelerated proliferation activity, decreased apoptosis, shortened G0/G1 phase and down-regulated GPX3 expression, all of which could be reversed by TRO treatment.

**Table 1:** Variation of biometric and physiological parameters in different treatment rats

Group	Body weight (g)		Ventral prostate weight (mg)	Seminal vesicles weight (mg)	Prostate index
	Initial	Final			
Con	267.4 ± 15.6	451.4 ± 24.3	597.2 ± 94.8	1144.1 ± 165.0	1.3 ± 0.2
T	259.8 ± 17.2	383.6 ± 17.8^***^	1028.9 ± 94.6^***^	2524.0 ± 288.6	2.7 ± 0.2^***^
T + RSL3	266.1 ± 15.7	335.9 ± 23.4	572.1 ± 102.9	1337.0 ± 118.4	1.7 ± 0.3
T + TRO	263.3 ± 14.8	429.1 ± 23.1	711.9 ± 83.7	1282.9 ± 187.6	1.7 ± 0.3
T + RSL3 + TRO	264.4 ± 16.2	408.7 ± 21.5	539.3 ± 53.6^ ns^	1276.9 ± 164.8	1.3 ± 0.2

*T* testosterone; *RSL3* RAS-selective lethal 3; *TRO* troglitazone

^***^p < 0.001 Con vs. T

*p* < 0.01 T vs. T + RSL3; *p* < 0.001 T vs. T + RSL3

*p* < 0.01 T vs. T + TRO; *p* < 0.001 T vs. T + TRO;

*p* < 0.05 T + RSL3 vs. T + RSL3 + TRO; *p* < 0.001 T + RSL3 vs. T + RSL3 + TRO

ns *p* > 0.05 T + RSL3 vs. T + RSL3 + TRO

**Fig. 11:**
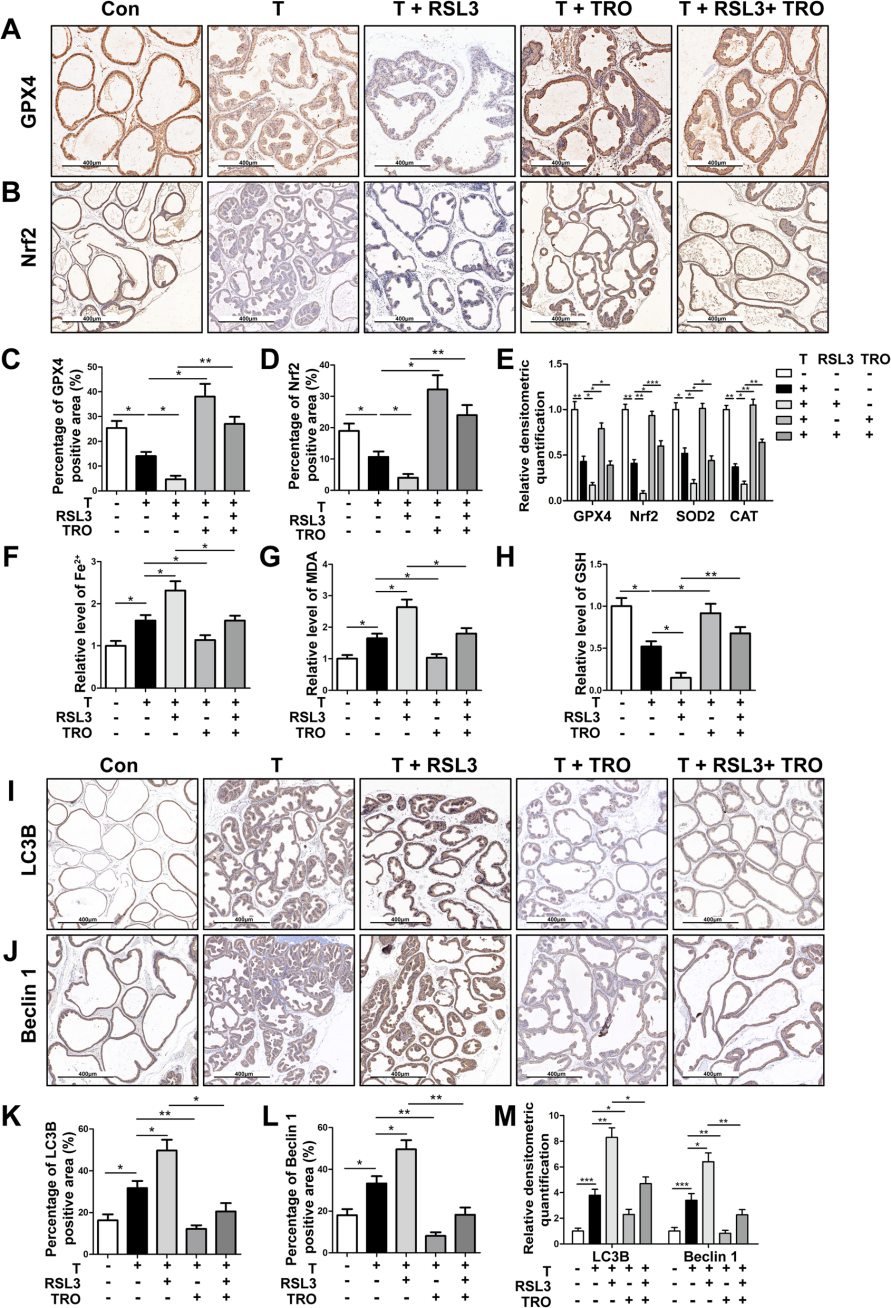
Effect of GPX3 on autophagy and ferroptosis of prostate in vivo. Representative IHC images of GPX4 **A** and Nrf2 **B** in prostate of Con, testosterone (T), T + RAS-selective lethal 3 (RSL3), T + troglitazone (TRO) and T + RSL3 + TRO-treated rats group. The quantitative analysis of GPX4 **C** and Nrf2 **D** expression in each group rat prostates. **E** Relative densitometric quantification of GPX4, Nrf2, SOD2 and CAT in prostate of each treatment group. The content of iron **F**, MDA **G** and GSH **H** in prostate of each treatment group were detected by colorimetric assay kit. The results indicated that the ferroptosis and OS levels in the prostate of T-BPH rats could be aggravated by RSL3 treatment, while inhibited by up-regulation of GPX3 in vivo*.* Representative IHC images of LC3B **I** and Beclin1 **J** in prostate of each treatment group. The quantitative analysis of LC3B **K** and Beclin1 **L** expression in each group rat prostates. **M** Relative densitometric quantification of LC3B and Beclin1 in prostate of each treatment group. The results showed that the autophagy level in the prostate of T-BPH rats could be aggravated by RSL3 treatment, while inhibited by up-regulation of GPX3 in vivo*.* ^*^p < 0.05, ^**^p < 0.01, ^***^p < 0.001. The scale bars are 400 μm

Also, IHC staining and Western blot results showed that the expression of GPX4 (Fig. 11A, C, E and Additional file 11: Fig. S7C) and Nrf2 (Fig. 11B, D, E and Additional file 11: Fig. S7C) decreased in T-BPH rat prostate and further decreased after RSL3 treatment, while could be rescued by TRO treatment. Furthermore, T treatment significantly increased the levels of iron (Fig. 11F) and MDA (Fig. 11G), while decreased the levels of SOD2, CAT (Fig. 11E and Additional file 11: Fig. S7C) and GSH (Fig. 11H) in the rat prostate, indicating that the level of ferroptosis in the prostate of T-induced BPH rats was elevated, which could be further aggravated by RSL3 (Fig. 11E–H). In addition, TRO treatment antagonized ferroptosis caused by T or RSL3 (Fig. 11E–H). Meanwhile, IHC staining and Western blot experiments suggested that T treatment could up-regulate the levels of LC3B (Fig. 11I, K, M and Additional file 11: Fig. S7D) and Beclin1 (Fig. 11J, L, M, Additional file 11: Fig. S7D) in T-BPH rat prostates, which could be exacerbated by RSL3 treatment and partially reversed by TRO. This indicated that the autophagy level of T-BPH rat prostates was relatively elevated and could be further aggravated by ferroptosis inducer (RSL3), while the up-regulation of GPX3 expression (TRO treatment) could significantly antagonize autophagy induced by T or RSL3.

## Discussion

**Fig. 12:**
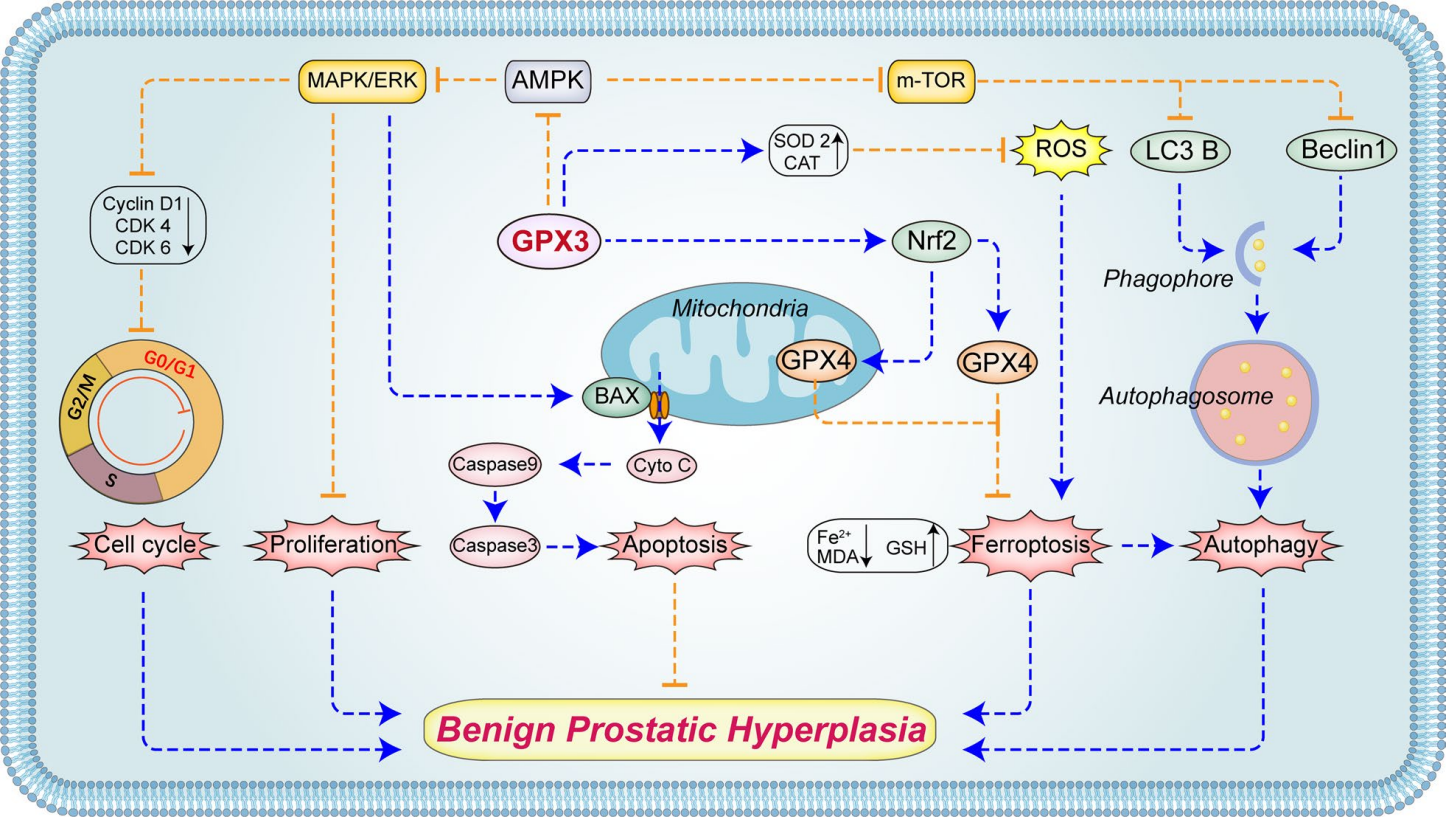
The mechanism of GPX3 on prostate cells. GPX3 inhibited AMPK activation in prostate cells, and subsequently restrained proliferation, promoted G0G1 phase arrest and mitochondrial-mediated apoptosis by up-regulating ERK1/2. On the other hand, autophagy-related ferroptosis was antagonized by activating m-TOR, accompanied by activation of Nrf2/GPX4 and up-regulation of antioxidant components (SOD2 and CAT). Due to various factors leading to downregulation of GPX3 expression, the above functions were reversed and involved in the occurrence and development of BPH. The blue arrow dotted line represents activation/promotion, and the orange blocking dotted line represents inhibition/antagonism

Our novel data demonstrated that GPX3 was localized in the stromal and epithelial compartments of prostate tissues and down-regulated in hyperplastic prostate tissues. Furthermore, we manifested that GPX3 not only had antioxidant properties, but also played roles in inhibiting AMPK pathway. Subsequently, it inhibited cell proliferation, promoted G0/G1 phase arrest and mitochondrial-dependent apoptosis via ERK1/2. On the other hand, it exerted anti-autophagy function by activating mTOR and antagonized autophagy-related ferroptosis mediated by Nrf2/GPX4 (Fig. 12).

GPX3 is an extracellular glycoprotein that accepts thioredoxin (Trx) and glutaredoxin (Grx) as substrates in addition to GSH [13]. As an extracellular antioxidant device, plasma GPX3 is mostly produced by the kidneys, while other organs such as lung, breast, adipose, epididymis and prostate, express GPX3 locally in cell type dependent manner [13]. It has been reported that GPX3 deficiency could enhance the cell proliferation of prostate cancer (PCa) tissues and reduce PCa cell apoptosis [38]. On the other hand, Yu et al. [20] showed that overexpression of GPX3 could inhibit the proliferation of PCa cells and the size of xenograft tumors in vivo*.* Interestingly, GPX3 activity progressively decreased with age, which was more significant for people over 70 years old. It had been reported that the age-related decline of GPX3 may increase the risk of cardiovascular events in patients with atrial fibrillation [39]. As we known, BPH is a common disease in aging male. And our previous microarray data did find the decrease of GPX3 in the aged prostate. Current study further validated that the expression of GPX3 in hyperplastic prostate was significantly lowered both at the transcriptional and translational levels. So did with the T-BPH rat model. However, the average age of healthy prostates and BPH prostates (25.2 ± 4.4 years vs. 70.0 ± 7.5 years) was significantly different, which seemed to be not conducive to distinguish the effect of aging on GPX3 expression. In fact, approximately 50% of men > 50 years of age will have pathological evidence of BPH, with this incidence increasing to > 80% as men reach their eighth decade of life or older [40], which means that we could hardly get healthy prostate samples from men over the age of 70 in existing clinical scenario. Comparing the down-regulated expression of GPX3 with age in healthy prostates and hyperplastic prostates around 70 years old will be of great significance for clarifying the expression pattern of GPX3 in prostate, which needs further study.

The imbalance between proliferation and apoptosis is one of the most important pathogenic mechanisms of BPH. In mammalian cells, Bcl-2 associated X protein (BAX) is recruited when various endogenous and exogenous stimuli induce apoptosis and form pores in the mitochondrial membrane [41], facilitating the release of Cyto-C (as a well conserved electron-transport protein and a part of the respiratory chain localized in mitochondrial intermembrane space.) into the cytoplasm. Cyto-C on the cytoplasm further activate caspase 9, which in turn triggers caspase 3 to initiate apoptosis [42]. Bcl-2 acts as an anti-apoptotic protein, counteracting BAX activation to maintain this balance of programmed cell death [43]. And the imbalance between proliferation and apoptosis is closely related to abnormal cell cycle regulation. Cyclin dependent kinases (CDKs) are a group of serine/threonine protein kinases. As the engine of cell cycle, CDKs and their regulatory factors exert an important effect in disease progression. CDK4 and CDK6 are important CDKs members that regulated the transition from G1 to S phase and bind to Cyclin Ds (D1, D2 and D3) to form CDK4/6-Cyclin complex after being activated by mitotic signals, while the over-activated CDK4/6 destabilizes the genome and chromosome, leading to uncontrolled proliferation and ultimately abnormal cell cycle regulation [44]. Moreover, CDK1-Cyclin B1 complex is involved in the regulation of cell cycle as an important checkpoint of G2/M phase transition [45]. In this study, we found GPX3 knocked-down shortened the G0/G1 phase, up-regulated the expression of Cyclin D1, CDK4 and CDK6, and arrested G2 phase, which seemed to contradict the accelerated cell proliferation and decreased apoptosis led by GPX3 silenced. Therefore, we combined si-GPX3s with isosilybin B, which plays a pro-apoptotic role by blocking the G1 phase [46], to detect the level of apoptosis. It was showed that the increase in apoptosis caused by isosilybin B could be significantly inhibited by GPX3 knockdown and GPX3 knockdown had no significant effect on the expression of Cyclin B1 and CDK1, which confirmed that the down-regulation of GPX3 regulated cell proliferation and apoptosis by shortening G0/G1 phase, while the prolongation of G2 phase seemed to be a compensatory change. Furthermore, we observed that overexpression of GPX3 could inhibit the proliferation of prostate cells, induce G0/G1 phase arrest, cell apoptosis, MMP dissipation and cytoplasmic transfer of Cyto-C. Specifically, overexpressed GPX3 induced mitochondrial dysfunction and cytoplasmic transfer of Cyto-C through up-regulation of BAX and down-regulation of Bcl-2, and ultimately induced mitochondria-dependent apoptosis via the caspase 9-caspase 3 cascade, and then cooperated with the blocking effect of GPX3 on G0/G1 phase to attenuate the proliferation activity of prostate cells. In addition, we also observed lower expression of BAX and caspase 3 and higher expression of Bcl-2 in hyperplastic prostate tissues compared with normal prostate tissues, which showed that with the down-regulation of GPX3, the apoptosis level of hyperplastic prostate was inhibited at the tissue level.

Three characteristic MAPK families (ERK, JNK and p38 kinases) have been identified in mammalian cells [47]. There was evidence that all three MAPK cascades might be involved in the progression of BPH by regulating the local prostate environment [48, 49]. Although in most reports, the ERK1/2 pathway has been shown to promote cell survival, proliferation and motility, it was also reported inhibited cell apoptosis. In the study of cisplatin-induced apoptosis of renal epithelial cells, Kim et al. [50] found that activated ERK1/2 played a pro-apoptotic role by inducing BAX expression, MMP depolarization, mitochondrial Cyto-C release and caspase3 upregulation, which was in line with our findings. In this study, when GPX3 was knocked down in prostate cells, the phosphorylation level of ERK1/2 was significantly inhibited with no significant effect on JNK1/2 and p38. In current study, prostate cells were further pre-incubated with MEK1/2 inhibitor U0126 and then transfected with overexpressed GPX3. We found that GPX3 overexpression could significantly up-regulate the phosphorylation of ERK1/2 but had a weak effect on JNK1/2 and p38. Meanwhile, U0126 could largely offset the effects of GPX3 overexpression on cell proliferation, cell cycle and apoptosis. Our data suggested that GPX3 triggered G0/G1 arrest and mitochondrial-dependent apoptosis through ERK1/2 activation rather than JNK1/2 and p38 in hyperplastic prostate. Furthermore, we found that the activity of ERK1/2 was negatively regulated by AMPK in vitro. AMPK is an energy sensor that monitors the AMP: ADP: ATP ratio in eukaryotic cells and conclusive evidence showed that AMPK could directly phosphorylate the Raf proto-oncogene/Kinase suppressor of Ras (RAF/KSR) family kinases, the pivotal components of MAPK module, and alter their activities under variable conditions [51, 52]. In addition to mediating ERK1/2 signaling, we showed that AMPK pathway could also participate in autophagy-related ferroptosis of prostate cells.

Ferroptosis is a newly identified form of nonapoptotic programmed cell death characterized by iron-dependent accumulation of lipid peroxides, and has drawn wide attention since the term was put forward in 2012 [25]. It is morphologically and biochemically different from known types of cell death. To be specific, the most notable morphological feature of ferroptotic cells under electron microscopy is the change in mitochondrial morphology; ferroptotic cells typically contain shrunken mitochondria with increased membrane density [53] and the main biochemical characteristics of ferroptosis consist of increased cellular labile iron, large amounts of ROS, decreased GPX4 activity, and accumulation of lipid metabolites [54]. In this study, we were the first to examine the ferroptosis level in hyperplastic prostate. We found that the expression levels of Nrf2 and GPX4 were decreased in both human hyperplastic prostates and T-induced BPH rat prostates. Meanwhile, the expression of CAT and SOD2, which are antioxidant components, was also at a low level in hyperplastic prostate tissues. It is suggested that Nrf2/GPX4 dysregulation-associated ferroptosis and high levels of OS occur in GPX3 down-regulated hyperplastic prostates. Furthermore, when GPX3 was knocked down, which was regarded as an antioxidant, endogenous ROS were undeniably elevated, and accompanied by downregulation of antioxidant proteins SOD2 and CAT. Meanwhile, the levels of GPX4 and its cofactor GSH decreased, while the markers of labile iron content (Fe^2+^) and lipid peroxidation product MDA increased significantly. Moreover, GPX3 deficiency also led to the downregulation of Nrf2, a negative upstream regulator of ferroptosis, suggesting that the antagonistic effect of GPX3 on ferroptosis in prostate cells was related to Nrf2. We further pretreated prostate cells with RSL3 and then transfected with overexpressed GPX3. RSL3, a compound containing an electrophilic moiety and a chloroacetamide moiety, could reacted with the nucleophilic amino acid residue at the active site of GPX4. This binding directly led to the inactivation of GPX4. We observed that RSL3 treatment significantly increased the accumulation of labile iron, ROS and lipid peroxides and accompanied by the reduction or disappearance of mitochondrial cristae in prostate cells, while GPX4 decreased. Whereas, GPX3 overexpression could largely reverse the effect of RSL3 on activating ferroptosis via upregulating Nrf2 and GPX4. To escape ferroptosis, cells evolve to be equipped with defending systems that detoxify lipid peroxidation and suppress ferroptosis. GPX4 [including cytosol- and mitochondria-localized GPX4 (GPX4^cyto^ and GPX4^mito^)] is the first reported anti-ferroptosis pathway [55]. Moreover, FSP1, which locates in the plasma membrane, prevents lipid peroxidation and inhibits ferroptosis even in the absence of GPX4 [56]. In addition, Mao et al. [57] reported that DHODH was an enzyme localized on the outer face of the mitochondrial inner membrane, which was functionally independent from GPX4^mito^, and both could detoxify lipid peroxides accumulated in mitochondria. In the present study we observed that when the expression level of GPX3 in prostate cells changed, both GPX4^mito^ and GPX4^cyto^ showed the consistent changes with GPX3, while FSP1 in the cytoplasm remained unchanged. However, when GPX4^mito^ was down-regulated with GPX3 knockdown, the expression level of DHODH in mitochondria increased, whereas GPX3 overexpression did not affect it. This could be attributed to the parallel effect of DHODH and GPX4^mito^ on inhibiting ferroptosis. When GPX4^mito^ was inactivated, DHODH occurred an adaptive cellular response that attempted to antagonize ferroptosis. In addition, we found that RSL3 not only induced ferroptosis in prostate cells, but also further promoted the increase of autophagy levels.

Autophagy is a highly conserved evolutionary and complex cellular process in eukaryotic cells, where cytoplasmic long half-life proteins and organelles are sequestered within autophagosomes and delivered to lysosomes for degradation and recycling [58]. Previous studies have revealed that autophagy was involved in BPH development [59, 60], and LC3B and Beclin1, as indicators of autophagy, were highly expressed in BPH stromal and epithelial tissues. Consistently, we found the high expression of LC3B and Beclin1 in hyperplastic prostate tissues and T-BPH rats, indicating that when the level of GPX3 in hyperplastic prostate tissues was down-regulated, it was accompanied by a high level of autophagy. Interestingly, there was a complex crosstalk between ferroptosis and autophagy through complex feedback loops [61, 62]. Ferroptosis induction was coupled to an increase in turnover of LC3B and autophagosome formation, consistent with the notion that lipid peroxidation as well as oxidized lipids could promote autophagy activation [63]. Similar to the discovery that RSL3 could promote autophagy activation in glioma cells [64]. In this study, RSL3 promoted autophagy activation in prostate cells, and this stimulation could be antagonized by GPX3 overexpression via down-regulating LC3B and Beclin1. However, functional contributions of GPX3 to autophagy-related ferroptosis have not been fully understood. The GPX3-mediated autophagic pathway could be modulated in a pharmacological approach. Ferroptosis in prostate cell lines could be aggravated by the autophagy activator Rapa, while the effects were reversed by the autophagy inhibitor CQ. Therefore, we inferred that GPX3-mediated autophagy may be an upstream mechanism that could adjust cellular OS and iron homeostasis during ferroptosis.

AMPK, as a key protein involved in various signal transduction pathways, activates autophagy through negative regulation of mTOR [37]. As expected, our data suggested that increased GPX3 exerted antagonistic effects on autophagy by down-regulating AMPK phosphorylation and up-regulating mTOR. In addition, AMPK agonist GSK621 up-regulated the protein levels of LC3B and Beclin1, and further increased the level of ferroptosis, which could be antagonized by GPX3 overexpression. These data demonstrated that GPX3 antagonized autophagy and the associated ferroptosis by inhibiting AMPK/mTOR. The function of AMPK in mediation of ferroptosis was required for Beclin1 phosphorylation, with inhibition of system Xc − activity being reported [65]. Perhaps AMPK could represented a mechanistic link between ferroptosis and autophagy. But its specific mechanism need be further studied.

Finally, we further translated our in vitro studies to in vivo. Since GPX3 recombinant protein or agonist was not commercially available, we used TRO gavage to mimic GPX3 overexpression [66] and intraperitoneal injection of RSL3 to initiate ferroptosis in vivo in rats. TRO is a ligand of peroxisome proliferator-activated receptor γ (PPARγ), which activates GPX3 as a direct upstream factor [67]. Our T-induced BPH model was verified with the dramatically augmented weight of the prostate and seminal vesicle when compared with control group. In line with previous observations, T injection mainly led to a notable thickening of the epithelial layer and collagen fiber with no significant change in the percentage of the SM component, which were features of T-BPH rats [36]. In addition, the prostate of T-BPH rats showed significantly accelerated proliferation activity, shortened cell cycle, decreased apoptosis, and up-regulated ferroptosis, OS and autophagy levels, which were consistent with what we observed in human hyperplastic prostate. Moreover, GPX3 overexpression (TRO treatment) could reverse the above T-induced phenotypic changes in rat prostate to a certain extent. In brief, all these changes were consistent with our in vitro findings. It should be noted that both RSL3 and TRO have inhibitory effects on the proliferation activity and epithelial thickness of the prostate in T-BPH rats (Fig. 10C–F), which seems unreasonable. In this in vivo study, the prostate of T-BPH rats treated with RSL3 showed significant increase in ferroptosis and autophagy, while TRO treatment showed a significant increase in apoptosis and G0/G1 phase arrest. This seems to indicate that the two have the same effect on the proliferation activity of rat prostates, but the mechanism is completely different, which needs further study.

Interestingly, our data observed that GPX3 had the pro-apoptosis but anti-autophagy-related ferroptosis effect in prostate cells. Study had shown that autophagy inhibition could enhance the apoptosis induced by androgen deprivation in human BPH-1 cells [68]. In this study, both in human prostate tissues and in the prostate of T-BPH rats, there were inhibition of apoptosis and abnormal activation of autophagy, as well as increased ferroptosis levels (in an autophagy-related manner). This seemed to suggest the protective role of autophagy and autophagy-related ferroptosis in BPH. Their specific mechanism in the occurrence and development of BPH needs to be further studied.

There are still some shortcomings in this study. Even if GPX4 was inhibited by RSL3, the ferroptosis phenotype was still effective under the context of GPX3 overexpression. Whether GPX3-mediated ferroptosis regulation of prostate cells works only through GPX4 is still unknown. Furthermore, all rescue experiments in this study used chemical inhibitors or agonists, which may have the risk of off-target effects. The use of genetic technology to regulate specific genes would strengthen the evidence. And the transgenic mice will further corroborate the role of GPX3 in the pathogenesis of BPH. These will be supplemented in future study.

## Conclusions

Our current study indicated that GPX3 protein was localized in both the stromal and epithelial compartments of prostate tissues with the transcription and translation levels of GPX3 were down-regulated in the hyperplastic prostate. Our novel data also demonstrated that GPX3 played double roles in the development of BPH in vivo and in vitro. On the one hand, GPX3 deficiency promoted cell proliferation, shortened G0/G1 phase and inhibited mitochondria-dependent apoptosis through AMPK/ERK1/2 pathway. On the other hand, GPX3 deficiency up-regulated autophagy-related ferroptosis via AMPK/mTOR pathway, and restrained Nrf2/GPX4 levels. Therefore, this study not only provided evidence for the future in-depth study of GPX3 in BPH, but also its multiple functional mechanisms were expected to become a new therapeutic target for BPH.

### Supplementary Information

**Additional file 1: Table S1.** Primer sequences used for qRT-PCR.

**Additional file 2: Table S2.** List of primary antibodies.

**Additional file 3 : Table S3.** List of secondary antibodies.

**Additional file 4: Table S4.** Sense sequences of siRNA.

**Additional file 5: Figure S1.** The heatmap plot of prostate DEGs (data from GSE119195 dataset). The heat map of 34 DEGs in 5 benign prostatic hyperplasia samples and 3 normal prostate samples. The legend color bar on the right side indicates the relation between scaled expression values and colors, and the red box highlights the GPX3 gene.

**Additional file 6: Figure S2.** Effect of GPX3 knockdown on GPX3 protein expression, cell cycle-related proteins, cell apoptosis, apoptosis-related proteins and MAPK pathway-related proteins in BPH-1 and WPMY-1 cells **A** Immunoblot assay of GPX3 in BPH-1 and WPMY-1 after knockdown of GPX3. **B** Immunoblot assay of Cyclin D1, CDK4, CDK6, Cyclin B1 and CDK1 in BPH-1 and WPMY-1 after knockdown of GPX3. The results showed that GPX3 silencing only affected G0/G1 checkpoint proteins but not G2/M checkpoint proteins expression.** C** The inhibition of proliferation by isosilybin B from 0 to 100 μM, the IC_50_ for the cytotoxic effect of BPH-1 and WPMY-1 cells was 67.72 and 41.38 μM, respectively.** D** Flow cytometry analysis of cell apoptosis. **E **Statistical analysis of apoptotic rate (%). Data showed that GPX3 silencing reversed the pro-apoptotic effect of isosilybin B, a G0/G1 phase apoptosis inducer, which confirmed GPX3 was involved in cell survival regulation by mediating G0/G1 phase. **F** The expression of Cyto-C in mitochondria or cytoplasm was detected after knockdown of GPX3. Tom 20 was detected as loading control for mitochondrial fraction. Tubulin was detected as loading control for cytosolic fraction. **G** Immunoblot assay of apoptosis-related proteins (Cleaved-Caspase 9, Cleaved-Caspase 3, Bcl-2 and BAX) in BPH-1 and WPMY -1 after knockdown of GPX3. **H** Immunoblot assay of MAPK signaling pathway proteins in BPH-1 and WPMY -1 after knockdown of GPX3. GAPDH is used as loading control. ^*^p < 0.05, ^**^p < 0.01.

**Additional file 7: Figure S3.** Effects of untreated group (con), transfected control (si-con) group and vector group on GPX3 expression, cell proliferation, cell cycle, apoptosis, MMP and MAPK pathway protein expression **A** Immunoblot assay of GPX3 in BPH-1 and WPMY-1 after different treatment. **B** Relative densitometric quantification of GPX3 protein in BPH-1 and WPMY-1 after different treatment. **C** The cell viability of BPH-1 and WPMY -1 after different treatment at different time points by CCK-8 assay; ^ns (red)^ con^ns (Biue)^ vs. si-con: con vs. vector. **D** Flow cytometry analysis of cell cycle. **E** Histogram showing percentage of cell populations at different stages of the cell cycle (%). **F** Flow cytometry analysis of cell apoptosis. **G** Statistical analysis of apoptotic rate (%). **H** The mitochondrial membrane potential level of BPH-1 and WPMY-1 cells was examined by JC-1 staining. The scatter plot of the flow cytometry analysis shows the distribution of JC-1 aggregates (Orange) and JC-1 monomer (Blue) cell population. **I** Histogram calculated the relative ratio of Orange against Blue fluorescence. **J** Immunoblot assay of MAPK signaling pathway proteins in BPH-1 and WPMY -1 after different treatment. **K** Relative densitometric quantification of MAPK signaling pathway proteins in BPH-1 and WPMY-1 after different treatment. GAPDH is used as loading control. ns means no significant difference.

**Additional file 8: Figure S4.** Effect of GPX3 overexpression and U0126 on cell cycle, apoptosis and MAPK signaling pathway proteins in prostate cells **A** Immunoblot assay showed the efficiency of GPX3 overexpression in prostate cells at the protein level. **B **Immunoblot assay of proteins in relation to cell cycle in BPH-1 and WPMY-1 after overexpression of GPX3.** C** The expression of Cyto-C in mitochondria or cytoplasm was detected after overexpression of GPX3. Tom 20 was detected as loading control for mitochondrial fraction. Tubulin was detected as loading control for cytosolic fraction. **D **Immunoblot assay of apoptosis-related proteins in BPH-1 and WPMY -1 after overexpression of GPX3. **E** Immunoblot assay of MAPK signaling pathway proteins in BPH-1 and WPMY -1 after overexpression of GPX3. **F** Immunoblot assay of apoptosis-related proteins (Cleaved-Caspase 9, Cleaved-Caspase 3, Bcl-2 and BAX) in BPH-1 and WPMY-1 after GPX3 overexpression or U0126 treatment. **G** Immunoblot assay of phosphorylated and total ERK1/2 as well as cell cycle related proteins (Cyclin D1, CDK4 and CDK6) in BPH-1 and WPMY-1 after GPX3 overexpression or U0126 treatment. GAPDH is used as loading control

**Additional file 9: Figure S5.** Effect of GPX3 expression on oxidative stress and ferroptosis-related protein expression in prostate cells **A** Immunoblot assay of proteins in relation to ferroptosis (GPX4 and Nrf2) and OS (SOD2 and CAT) in BPH-1 and WPMY-1 after knockdown of GPX3. **B** The expression of GPX4, FSP1 and DHODH in mitochondria or cytoplasm were detected after knockdown of GPX3. Tom 20 was detected as loading control for mitochondrial fraction. Tubulin was detected as loading control for cytosolic fraction.** C** The inhibition of proliferation by RSL3 from 0 to 4  μM, the IC_50_ for the cytotoxic effect of BPH-1 and WPMY-1 cells was 0.71 and 0.75  μM, respectively.** D** Immunoblot assay of proteins in relation to ferroptosis (GPX4 and Nrf2) and OS (SOD2 and CAT) in BPH-1 and WPMY-1 cells by GPX3 overexpression or RSL3 treatment. **E** The expression of GPX4, FSP1 and DHODH in mitochondria or cytoplasm was detected after overexpression of GPX3. Tom 20 was detected as loading control for mitochondrial fraction. Tubulin was detected as loading control for cytosolic fraction. GAPDH is used as loading control.

**Additional file 10: Figure S6.** Effect of GPX3 overexpression on RSL3-mediated autophagy and AMPK-mTOR pathway proteins **A** Immunoblot assay of proteins in relation to autophagy (LC3B and Beclin1) in BPH-1 and WPMY-1 cells treated with GPX3 overexpression or RSL3. **B **The inhibition of proliferation by Rapa from 0 to 8  nM, and the IC_50_ for the cytotoxic effect of BPH-1 and WPMY-1 cells was 2.67 and 3.24  nM, respectively. **C** The inhibition of proliferation by CQ from 0 to 50  μM, and the IC_50_ for the cytotoxic effect of BPH-1 and WPMY-1 cells was 28.52 and 31.04  μM, respectively. **D** Immunoblot assay of autophagy associated proteins (LC3B and Beclin1) in RSL3-pretreated BPH-1 and WPMY-1 cells after GPX3 overexpression, CQ or Rapa treatment. **E** Immunoblot assay of proteins (AMPK, p-AMPK, mTOR and p-mTOR) in BPH-1 and WPMY-1 cells treated with RSL3 (0.5 μM) or GPX3 plasmid. **F** The inhibition of proliferation by GSK621 from 0 to 40  μM, and the IC_50_ for the cytotoxic effect of BPH-1 and WPMY-1 cells was 23.21 and 25.33  μM, respectively. **G** Immunoblot assay of AMPK and p-AMPK in BPH-1 and WPMY-1 cells treated with 20 μM GSK621. **H** Immunoblot assay of proteins (LC3B and Beclin1) in RSL3-pretreated BPH-1 and WPMY-1 cells treated with GPX3 overexpression or GSK621 (20 μM). GAPDH is used as loading control.

**Additional file 11: Figure S7.** Effect of GPX3 on cell cycle, apoptosis, ferroptosis and autophagy of prostate in vivo* A* Immunoblot assay of GPX3 protein in prostate of each treatment group. **B** Immunoblot assay of Cleaved-Caspase 9, Cleaved-Caspase 3, Bcl-2, BAX, Cyclin D1, CDK4 and CDK6 in prostate of Con, T, and T + TRO-treatment group. **C** Immunoblot assay of GPX4, Nrf2, SOD2 and CAT in prostate of each treatment group. **D** Immunoblot assay of LC3B and Beclin1 in prostate of each treatment group. GAPDH is used as loading control.

## Data Availability

The data used to support the findings of this study are available from the corresponding author upon reasonable request.

## Abbreviations

BPH: Benign prostatic hyperplasia
LUTSs: Lower urinary tract symptoms
OS: Oxidative stress
DEGs: Differentially expressed genes
MXRA5: Matrix-remodeling associated 5
CXCL13: C-X-C Motif Chemokine Ligand 13
BMP5: Bone morphogenetic protein 5
NELL2: Neural epidermal growth factor-like like 2
GPX3: Glutathione peroxidase 3
GSH: Glutathione
ROS: Reactive oxygen species
Cyto-C: Cytochrome c
MMP: Mitochondrial membrane potential
PCD: Programmed cell death
MDA: Malondialdehyde
H&E: Hematoxylin and eosin staining
IHC: Immunohistochemical
DMSO: Dimethyl sulfoxide
T: Testosterone propionate
RSL3: RAS-selective lethal 3
GPX4: Glutathione peroxidase 4
TRO: Troglitazone
PPARγ: Peroxisome proliferator-activated receptor γ
Rapa: Rapamycin
CQ: Chloroquine
Nrf2: Nuclear factor erythroid 2-related factor 2
SOD2: Superoxide dismutase 2. CAT: catalase
DHODH: Dihydroorotate dehydrogenase
FSP1: Ferroptosis suppressor protein 1
TEM: Transmission electron microscopy
AMPK: Adenosine monophosphate-activated protein kinase
M-TOR: Mammalian target of rapamycin
PCa: Prostate cancer
