# Supplementary material for: Glutathione Peroxidase 3 induced mitochondria-mediated apoptosis via AMPK /ERK1/2 pathway and resisted autophagy-related ferroptosis via AMPK/mTOR pathway in hyperplastic prostate
Source: J Transl Med. 2023 Aug 26;21:575. doi: 10.1186/s12967-023-04432-9 (PMC10463608; doi:10.1186/s12967-023-04432-9)
Supplement: Supplementary file 1 — Additional file 1: Table S1. Primer sequences used for qRT-PCR. [file 12967_2023_4432_MOESM1_ESM.docx]

**Table S1. Primer sequences used for qRT-PCR**

| Target gene | | Human (5’ to 3’) | Tm |
| --- | --- | --- | --- |
| GPX3 | Forward | GAGCTTGCACCATTCGGTCT | 62.5 |
|  | Reverse | GGGTAGGAAGGATCTCTGAGTTC | 60.9 |
| GAPDH | Forward | GGAGCGAGATCCCTCCAAAAT | 61.6 |
|  | Reverse | GGCTGTTGTCATACTTCTCATGG | 60.9 |
