# Supplementary material for: Glutathione Peroxidase 3 induced mitochondria-mediated apoptosis via AMPK /ERK1/2 pathway and resisted autophagy-related ferroptosis via AMPK/mTOR pathway in hyperplastic prostate
Source: J Transl Med. 2023 Aug 26;21:575. doi: 10.1186/s12967-023-04432-9 (PMC10463608; doi:10.1186/s12967-023-04432-9)
Supplement: Supplementary file 2 — Additional file 2: Table S2. List of primary antibodies. [file 12967_2023_4432_MOESM2_ESM.docx]

**Table S2. List of primary antibodies**

| Antigens | Species antibodies  raised in | Dilution used | Supplier |
| --- | --- | --- | --- |
| GPX3 | Rabbit, polyclonal | 1:1000 (WB)  1:500 (IF)  1:500 (IHC) | Abbkine, CHN, Cat. ABP58707 |
| GAPDH | Rabbit, polyclonal | 1:1000 (WB) | Abclonal, CHN, Cat. AC001 |
| CDK4 | Rabbit, monoclonal | 1:1000 (WB) | CST, USA, Cat. 12790 |
| Cyclin D1 | Rabbit, monoclonal | 1:1000 (WB) | CST, USA, Cat. 2978 |
| CDK6 | Rabbit, monoclonal | 1:1000 (WB) | Abclonal, CHN, Cat. A0106 |
| Cyto-C | Rabbit, monoclonal | 1:1000 (WB) | Abclonal, CHN, Cat. A4912 |
| Tom 20 | Rabbit, monoclonal | 1:1000 (WB) | Abclonal, CHN, Cat. A19403 |
| Tubulin | Rabbit, polyclonal | 1:1000 (WB) | Abclonal, CHN, Cat. A17074 |
| Bcl-2 | Rabbit, polyclonal | 1:1000 (WB)  1:100 (IHC) | Abclonal, CHN, Cat. A11025 |
| BAX | Rabbit, polyclonal | 1:1000 (WB)  1:100 (IHC) | Abclonal, CHN, Cat. A12009 |
| Caspase 3 | Rabbit, polyclonal | 1:1000 (WB)  1:100 (IHC) | Abclonal, CHN, Cat. A2156 |
| Caspase 9 | Rabbit, monoclonal | 1:1000 (WB) | Abclonal, CHN, Cat. A11910 |
| ERK1/2 | Rabbit, monoclonal | 1:1000 (WB) | Abclonal, CHN, Cat. A4782 |
| p-ERK1/2 | Rabbit, polyclonal | 1:1000 (WB) | Abclonal, CHN, Cat. AP0472 |
| JNK1/2 | Rabbit, monoclonal | 1:1000 (WB) | Abclonal, CHN, Cat. A11119 |
| p- JNK1/2 | Rabbit, polyclonal | 1:1000 (WB) | Abclonal, CHN, Cat. AP0473 |
| P38 | Rabbit, monoclonal | 1:1000 (WB) | CST, USA, Cat. 8690 |
| p-p38 | Rabbit, monoclonal | 1:1000 (WB) | CST, USA, Cat. 4511 |
| GPX4 | Mouse, monoclonal | 1:1000 (WB)  1:1000 (IHC) | Proteintech, USA, Cat. 67763-1-Ig |
| NRF2 | Rabbit, monoclonal | 1:1000 (WB)  1:500 (IHC) | Abclonal, CHN, Cat. A21176 |
| SOD2 | Rabbit, monoclonal | 1:1000 (WB)  1:100 (IHC) | Abclonal, CHN, Cat. A19576 |
| CAT | Rabbit, monoclonal | 1:1000 (WB)  1:100 (IHC) | Abclonal, CHN, Cat. A11220 |
| FSP1 | Rabbit, polyclonal | 1:1000 (WB) | Abclonal, CHN, Cat. A1631 |
| DHODH | Rabbit, polyclonal | 1:1000 (WB) | Abclonal, CHN, Cat. A13295 |
| LC3B | Rabbit, polyclonal | 1:1000 (WB)  1:500 (IHC) | Abbkine, CHN, Cat. ABP57204 |
| Beclin 1 | Rabbit, polyclonal | 1:1000 (WB)  1:500 (IHC) | Abclonal, CHN, Cat. A11761 |
| AMPK | Rabbit, polyclonal | 1:1000 (WB) | Abclonal, CHN, Cat. A17290 |
| p-AMPK | Rabbit, polyclonal | 1:1000 (WB) | Abclonal, CHN, Cat. AP0883 |
| mTOR | Rabbit, polyclonal | 1:1000 (WB) | Abclonal, CHN, Cat. A2445 |
| p-mTOR | Rabbit, monoclonal | 1:1000 (WB) | Abclonal, CHN, Cat. AP0115 |
| Ki-67 | Rabbit, polyclonal | 1:1200 (IF) | Servicebio, CHN, Cat. GB111141 |
| Cyclin B1 | Rabbit, monoclonal | 1:1000 (WB) | Abclonal, CHN, Cat. A22435 |
| CDK1 | Rabbit, monoclonal | 1:1000 (WB) | Abclonal, CHN, Cat. A11420 |
