# Supplementary material for: Glutathione Peroxidase 3 induced mitochondria-mediated apoptosis via AMPK /ERK1/2 pathway and resisted autophagy-related ferroptosis via AMPK/mTOR pathway in hyperplastic prostate
Source: J Transl Med. 2023 Aug 26;21:575. doi: 10.1186/s12967-023-04432-9 (PMC10463608; doi:10.1186/s12967-023-04432-9)
Supplement: Supplementary file 3 — Additional file 3 : Table S3. List of secondary antibodies. [file 12967_2023_4432_MOESM3_ESM.docx]

**Table S3. List of secondary antibodies**

| Secondary detection system used | Host | Dilution used | Supplier |
| --- | --- | --- | --- |
| Anti-Mouse-IgG (H+L)-HRP | Goat | 1:10000 (WB) | Sungene Biotech, China, Cat. #LK2003 |
| Anti-Rabbit-IgG (H+L)-HRP | Goat | 1:10000 (WB) | Sungene Biotech, China, Cat. #LK2001 |
| Anti‐rabbit IgG (H + L), F(ab')2 fragment (Alexa Fluor®488 Conjugate) | Goat | 1:50 (IF) | Cell Signaling Technology, Cat. #4412 |
| 4′,6-diamidino-2-phenylindole (DAPI) | - | 1:750 (IF) | Molecular Probes/Invitrogen, Carlsbad, CA, USA, Cat. A11007 |
