# Supplementary material for: Glutathione Peroxidase 3 induced mitochondria-mediated apoptosis via AMPK /ERK1/2 pathway and resisted autophagy-related ferroptosis via AMPK/mTOR pathway in hyperplastic prostate
Source: J Transl Med. 2023 Aug 26;21:575. doi: 10.1186/s12967-023-04432-9 (PMC10463608; doi:10.1186/s12967-023-04432-9)
Supplement: Supplementary file 4 — Additional file 4: Table S4. Sense sequences of siRNA. [file 12967_2023_4432_MOESM4_ESM.docx]

**Table S4. Sense sequences of siRNA**

| Symbol | | | (5’ to 3’) |
| --- | --- | --- | --- |
| si-con | Sense sequence | UUCUCCGAACGUGUCAG UGACAUUAAGAUUCAGGGUTT | |
| si-GPX3-1 | Sense sequence | GGAACCACCAACUGACAAUTTAUUGUCAGUUGGUGGUUCCTT | |
| si-GPX3-2 | Sense sequence | GAAGGUGAUUGCCAGGAAATTUUUCCUGGCAAUCACCUUCTT | |
| si-GPX3-3 | Sense sequence | CCUAGGAAAUUGACACUAUTTAUAGUGUCAAUUUCCUAGGTT | |
