# Supplementary figures and images for: Glutathione Peroxidase 3 induced mitochondria-mediated apoptosis via AMPK /ERK1/2 pathway and resisted autophagy-related ferroptosis via AMPK/mTOR pathway in hyperplastic prostate
Source: J Transl Med. 2023 Aug 26;21:575. doi: 10.1186/s12967-023-04432-9 (PMC10463608; doi:10.1186/s12967-023-04432-9)

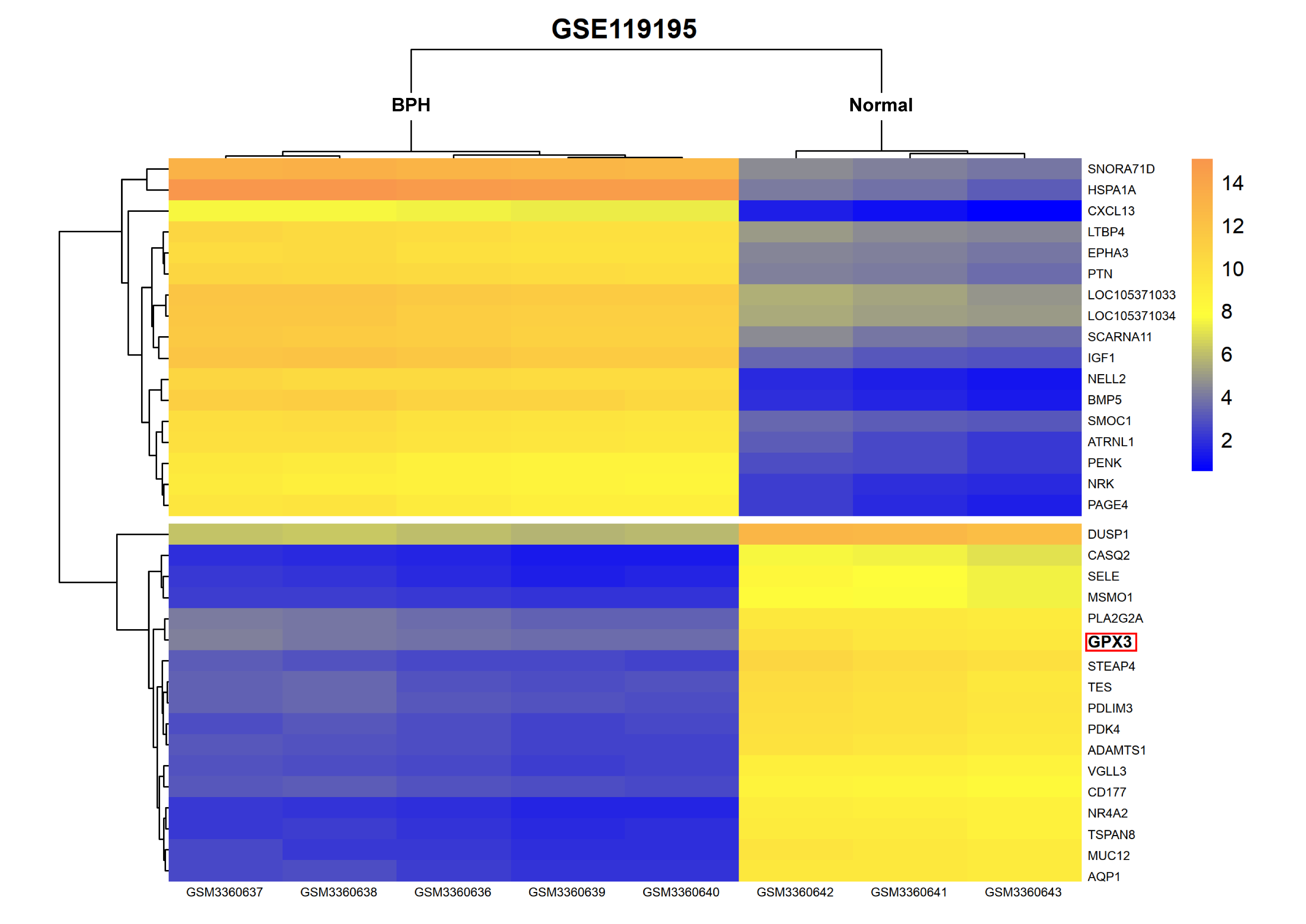

Supplement: Supplementary file 5 — Additional file 5: Figure S1. The heatmap plot of prostate DEGs (data from GSE119195 dataset). The heat map of 34 DEGs in 5 benign prostatic hyperplasia samples and 3 normal prostate samples. The legend color bar on the right side indicates the relation between scaled expression values and colors, and the red box highlights the GPX3 gene. [file 12967_2023_4432_MOESM5_ESM.tif]

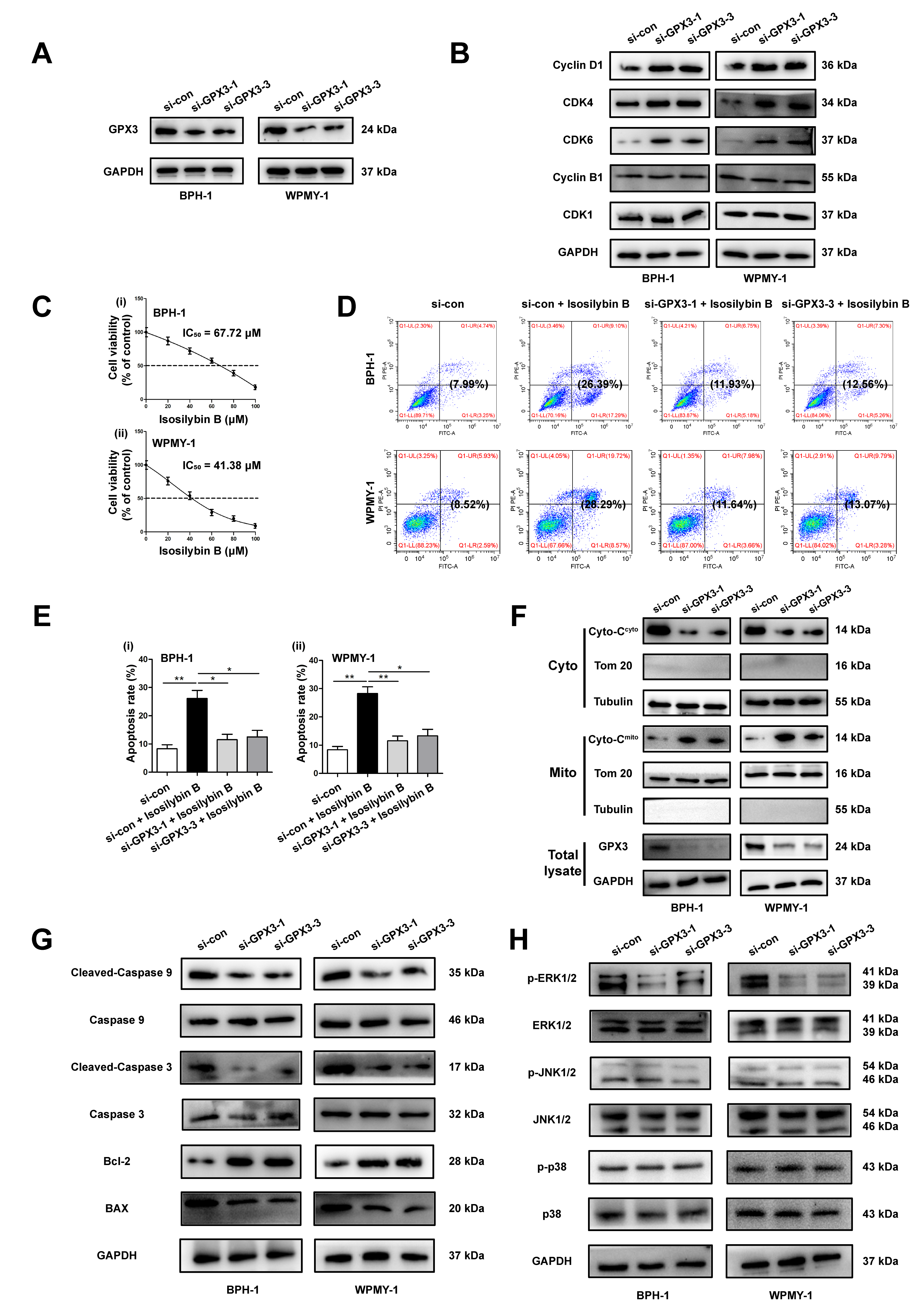

Supplement: Supplementary file 6 — Additional file 6: Figure S2. Effect of GPX3 knockdown on GPX3 protein expression, cell cycle-related proteins, cell apoptosis, apoptosis-related proteins and MAPK pathway-related proteins in BPH-1 and WPMY-1 cells A Immunoblot assay of GPX3 in BPH-1 and WPMY-1 after knockdown of GPX3. B Immunoblot assay of Cyclin D1, CDK4, CDK6, Cyclin B1 and CDK1 in BPH-1 and WPMY-1 after knockdown of GPX3. The results showed that GPX3 silencing only affected G0/G1 checkpoint proteins but not G2/M checkpoint proteins expression. C The inhibition of proliferation by isosilybin B from 0 to 100 μM, the IC50 for the cytotoxic effect of BPH-1 and WPMY-1 cells was 67.72 and 41.38 μM, respectively. D Flow cytometry analysis of cell apoptosis. E Statistical analysis of apoptotic rate (%). Data showed that GPX3 silencing reversed the pro-apoptotic effect of isosilybin B, a G0/G1 phase apoptosis inducer, which confirmed GPX3 was involved in cell survival regulation by mediating G0/G1 phase. F The expression of Cyto-C in mitochondria or cytoplasm was detected after knockdown of GPX3. Tom 20 was detected as loading control for mitochondrial fraction. Tubulin was detected as loading control for cytosolic fraction. G Immunoblot assay of apoptosis-related proteins (Cleaved-Caspase 9, Cleaved-Caspase 3, Bcl-2 and BAX) in BPH-1 and WPMY -1 after knockdown of GPX3. H Immunoblot assay of MAPK signaling pathway proteins in BPH-1 and WPMY -1 after knockdown of GPX3. GAPDH is used as loading control. *p < 0.05, **p < 0.01. [file 12967_2023_4432_MOESM6_ESM.tif]

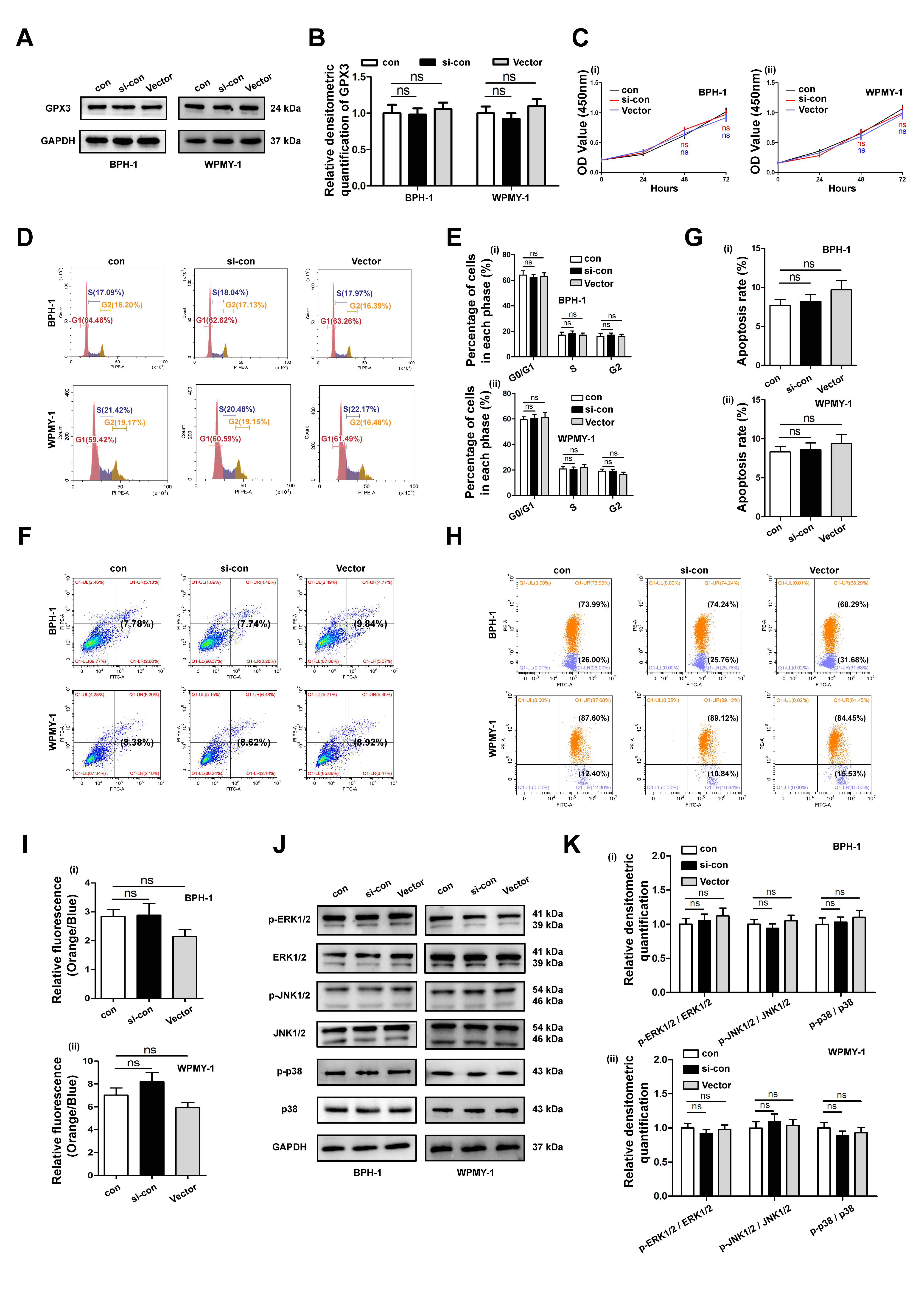

Supplement: Supplementary file 7 — Additional file 7: Figure S3. Effects of untreated group (con), transfected control (si-con) group and vector group on GPX3 expression, cell proliferation, cell cycle, apoptosis, MMP and MAPK pathway protein expression A Immunoblot assay of GPX3 in BPH-1 and WPMY-1 after different treatment. B Relative densitometric quantification of GPX3 protein in BPH-1 and WPMY-1 after different treatment. C The cell viability of BPH-1 and WPMY -1 after different treatment at different time points by CCK-8 assay; ns (red) conns (Biue) vs. si-con: con vs. vector. D Flow cytometry analysis of cell cycle. E Histogram showing percentage of cell populations at different stages of the cell cycle (%). F Flow cytometry analysis of cell apoptosis. G Statistical analysis of apoptotic rate (%). H The mitochondrial membrane potential level of BPH-1 and WPMY-1 cells was examined by JC-1 staining. The scatter plot of the flow cytometry analysis shows the distribution of JC-1 aggregates (Orange) and JC-1 monomer (Blue) cell population. I Histogram calculated the relative ratio of Orange against Blue fluorescence. J Immunoblot assay of MAPK signaling pathway proteins in BPH-1 and WPMY -1 after different treatment. K Relative densitometric quantification of MAPK signaling pathway proteins in BPH-1 and WPMY-1 after different treatment. GAPDH is used as loading control. ns means no significant difference. [file 12967_2023_4432_MOESM7_ESM.tif]

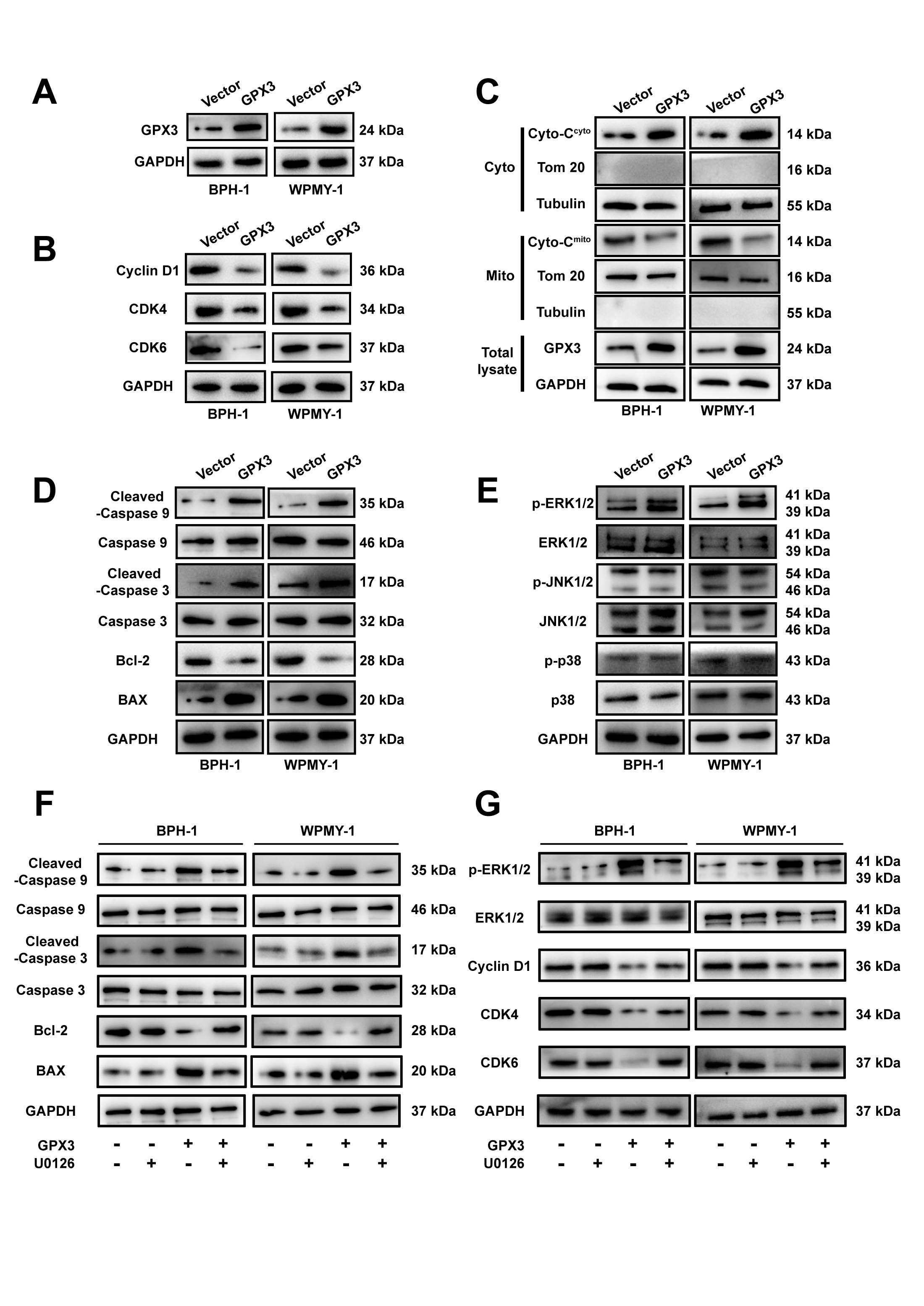

Supplement: Supplementary file 8 — Additional file 8: Figure S4. Effect of GPX3 overexpression and U0126 on cell cycle, apoptosis and MAPK signaling pathway proteins in prostate cells A Immunoblot assay showed the efficiency of GPX3 overexpression in prostate cells at the protein level. B Immunoblot assay of proteins in relation to cell cycle in BPH-1 and WPMY-1 after overexpression of GPX3. C The expression of Cyto-C in mitochondria or cytoplasm was detected after overexpression of GPX3. Tom 20 was detected as loading control for mitochondrial fraction. Tubulin was detected as loading control for cytosolic fraction. D Immunoblot assay of apoptosis-related proteins in BPH-1 and WPMY -1 after overexpression of GPX3. E Immunoblot assay of MAPK signaling pathway proteins in BPH-1 and WPMY -1 after overexpression of GPX3. F Immunoblot assay of apoptosis-related proteins (Cleaved-Caspase 9, Cleaved-Caspase 3, Bcl-2 and BAX) in BPH-1 and WPMY-1 after GPX3 overexpression or U0126 treatment. G Immunoblot assay of phosphorylated and total ERK1/2 as well as cell cycle related proteins (Cyclin D1, CDK4 and CDK6) in BPH-1 and WPMY-1 after GPX3 overexpression or U0126 treatment. GAPDH is used as loading control [file 12967_2023_4432_MOESM8_ESM.tif]

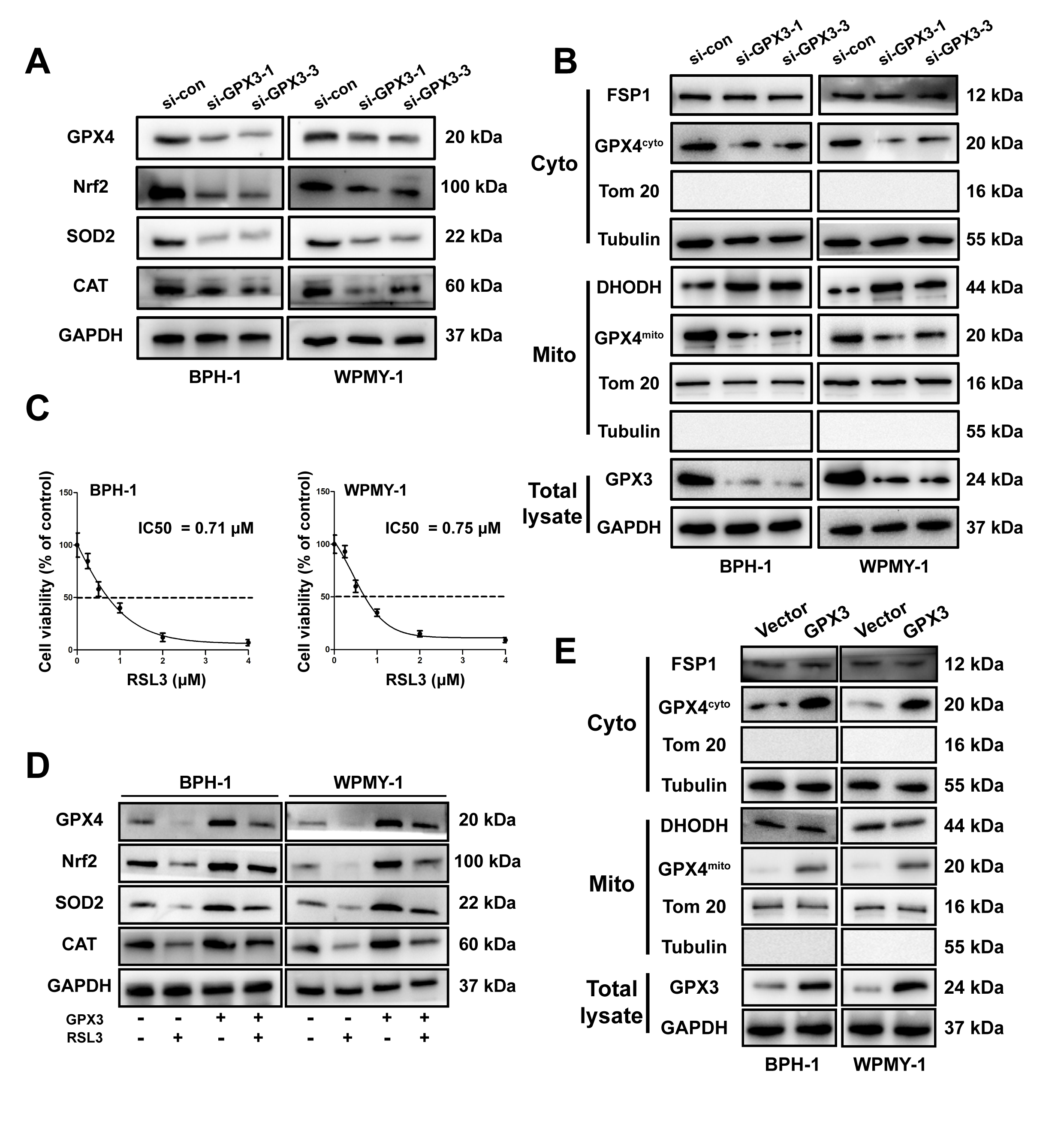

Supplement: Supplementary file 9 — Additional file 9: Figure S5. Effect of GPX3 expression on oxidative stress and ferroptosis-related protein expression in prostate cells A Immunoblot assay of proteins in relation to ferroptosis (GPX4 and Nrf2) and OS (SOD2 and CAT) in BPH-1 and WPMY-1 after knockdown of GPX3. B The expression of GPX4, FSP1 and DHODH in mitochondria or cytoplasm were detected after knockdown of GPX3. Tom 20 was detected as loading control for mitochondrial fraction. Tubulin was detected as loading control for cytosolic fraction. C The inhibition of proliferation by RSL3 from 0 to 4 μM, the IC50 for the cytotoxic effect of BPH-1 and WPMY-1 cells was 0.71 and 0.75 μM, respectively. D Immunoblot assay of proteins in relation to ferroptosis (GPX4 and Nrf2) and OS (SOD2 and CAT) in BPH-1 and WPMY-1 cells by GPX3 overexpression or RSL3 treatment. E The expression of GPX4, FSP1 and DHODH in mitochondria or cytoplasm was detected after overexpression of GPX3. Tom 20 was detected as loading control for mitochondrial fraction. Tubulin was detected as loading control for cytosolic fraction. GAPDH is used as loading control. [file 12967_2023_4432_MOESM9_ESM.tif]

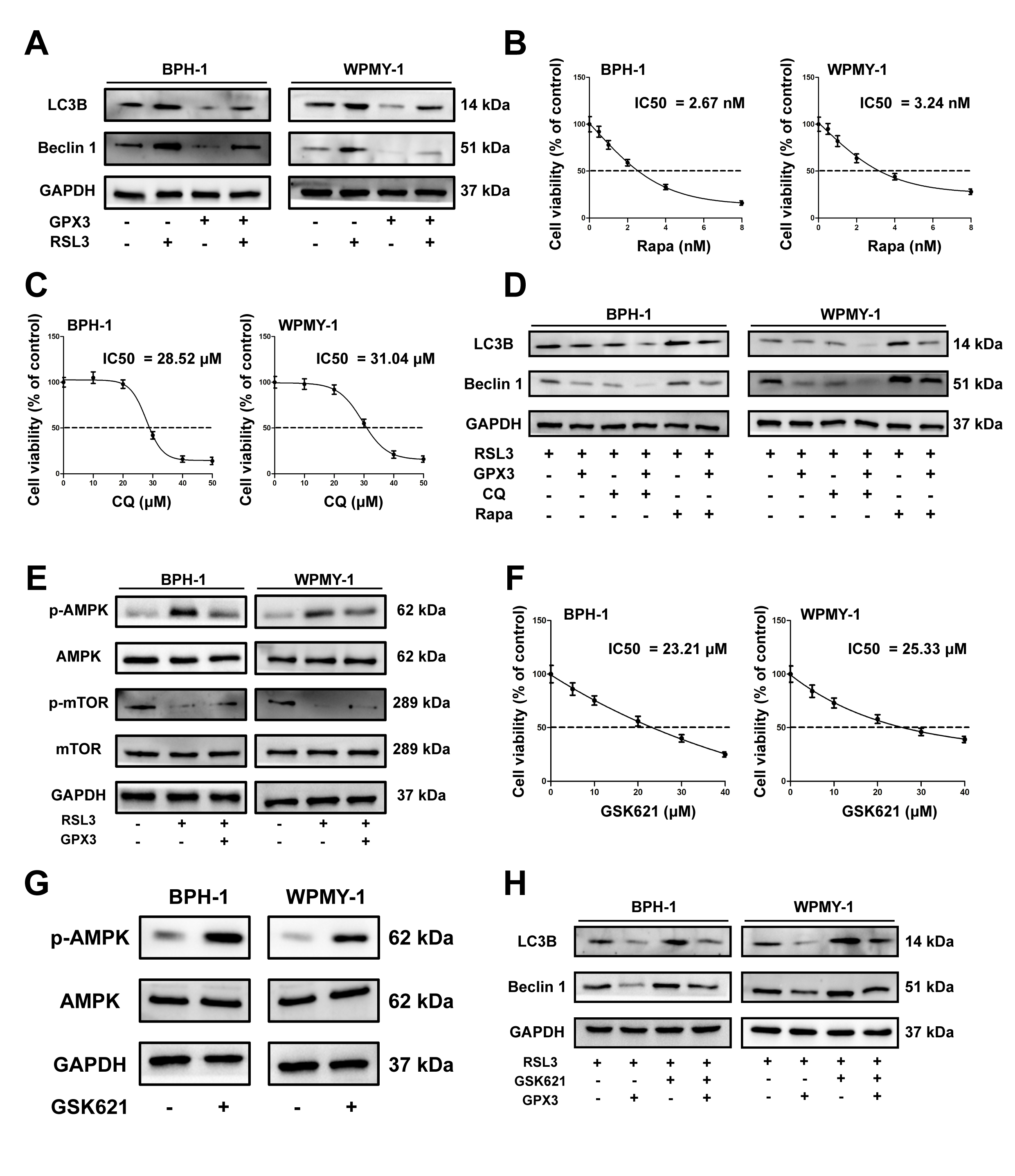

Supplement: Supplementary file 10 — Additional file 10: Figure S6. Effect of GPX3 overexpression on RSL3-mediated autophagy and AMPK-mTOR pathway proteins A Immunoblot assay of proteins in relation to autophagy (LC3B and Beclin1) in BPH-1 and WPMY-1 cells treated with GPX3 overexpression or RSL3. B The inhibition of proliferation by Rapa from 0 to 8 nM, and the IC50 for the cytotoxic effect of BPH-1 and WPMY-1 cells was 2.67 and 3.24 nM, respectively. C The inhibition of proliferation by CQ from 0 to 50 μM, and the IC50 for the cytotoxic effect of BPH-1 and WPMY-1 cells was 28.52 and 31.04 μM, respectively. D Immunoblot assay of autophagy associated proteins (LC3B and Beclin1) in RSL3-pretreated BPH-1 and WPMY-1 cells after GPX3 overexpression, CQ or Rapa treatment. E Immunoblot assay of proteins (AMPK, p-AMPK, mTOR and p-mTOR) in BPH-1 and WPMY-1 cells treated with RSL3 (0.5 μM) or GPX3 plasmid. F The inhibition of proliferation by GSK621 from 0 to 40 μM, and the IC50 for the cytotoxic effect of BPH-1 and WPMY-1 cells was 23.21 and 25.33 μM, respectively. G Immunoblot assay of AMPK and p-AMPK in BPH-1 and WPMY-1 cells treated with 20 μM GSK621. H Immunoblot assay of proteins (LC3B and Beclin1) in RSL3-pretreated BPH-1 and WPMY-1 cells treated with GPX3 overexpression or GSK621 (20 μM). GAPDH is used as loading control. [file 12967_2023_4432_MOESM10_ESM.tif]

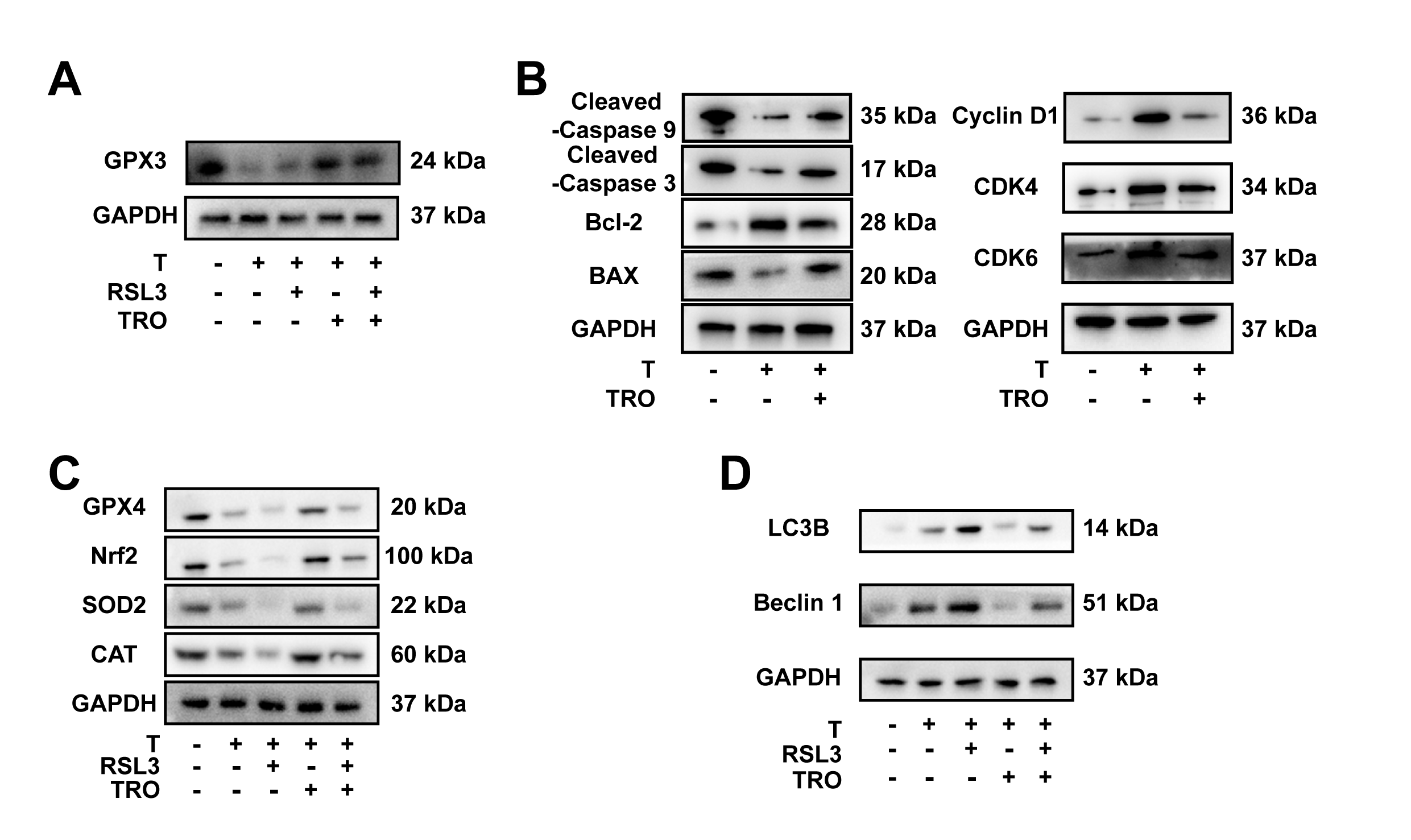

Supplement: Supplementary file 11 — Additional file 11: Figure S7. Effect of GPX3 on cell cycle, apoptosis, ferroptosis and autophagy of prostate in vivo A Immunoblot assay of GPX3 protein in prostate of each treatment group. B Immunoblot assay of Cleaved-Caspase 9, Cleaved-Caspase 3, Bcl-2, BAX, Cyclin D1, CDK4 and CDK6 in prostate of Con, T, and T + TRO-treatment group. C Immunoblot assay of GPX4, Nrf2, SOD2 and CAT in prostate of each treatment group. D Immunoblot assay of LC3B and Beclin1 in prostate of each treatment group. GAPDH is used as loading control. [file 12967_2023_4432_MOESM11_ESM.tif]
